# The role of thermal physiology in recent declines of birds in a biodiversity hotspot

**DOI:** 10.1093/conphys/cov048

**Published:** 2015-11-13

**Authors:** Robyn Milne, Susan J Cunningham, Alan T K Lee, Ben Smit

**Affiliations:** af1 Percy FitzPatrick Institute of African Ornithology, DST-NRF Centre of Excellence, University of Cape Town, Rondebosch 7701, South Africa; af2 Birds and Environmental Change Programme, Climate Change and Adaptation Division, South African National Biodiversity Institute, Claremont 7735, South Africa; af3 Centre for African Conservation Ecology, Department of Zoology, Nelson Mandela Metropolitan University, Port Elizabeth 6031, South Africa

**Keywords:** Bioclimatic envelope models, climate change, endemism, evaporative water loss, Fynbos, heat tolerance

## Abstract

We investigated whether observed avian range contractions and population declines in the Fynbos biome of South Africa were mechanistically linked to recent climate warming. We aimed to determine whether there were correlations between preferred temperature envelope, or changes in temperature within species' ranges, and recent changes in range and population size, for 12 Fynbos-resident bird species, including six that are endemic to the biome. We then measured the physiological responses of each species at air temperatures ranging from 24 to 42°C to determine whether physiological thermal thresholds could provide a mechanistic explanation for observed population trends. Our data show that Fynbos-endemic species occupying the coolest regions experienced the greatest recent reductions in range and population size (>30% range reduction between 1991 and the present). In addition, species experiencing the largest increases in air temperature within their ranges showed the greatest declines. However, evidence for a physiological mechanistic link between warming and population declines was equivocal, with only the larger species showing low thermal thresholds for their body mass, compared with other birds globally. In addition, some species appear more vulnerable than others to air temperatures in their ranges above physiological thermal thresholds. Of these, the high-altitude specialist Cape rockjumper (*Chaetops frenatus*) seems most at risk from climate warming. This species showed: (i) the lowest threshold for increasing evaporative water loss at high temperatures; and (ii) population declines specifically in those regions of its range recording significant warming trends. Our findings suggest that caution must be taken when attributing causality explicitly to thermal stress, even when population trends are clearly correlated with rates of warming. Studies explicitly investigating the mechanisms underlying such correlations will be key to appropriate conservation planning.

## Teaser

We integrated population, climate, and physiological data to investigate Fynbos birds' vulnerability to recent climate change. Species occupying cool ranges with warming trends showed greatest declines. However, physiological thermal thresholds varied and, with the exception of Cape Rockjumper, were not clearly the cause of decline in most species.

## Introduction

Anthropogenic climate change is recognized as one of the most important current threats to global biodiversity, with effects experienced in almost every biome (Thomas *et al.*, 2004; Millennium Ecosystem Assessment, 2005). Assessing the vulnerability of species to climate change is therefore of major importance for conservation biologists.

Bioclimatic envelope models are often used to predict future availability and distribution of suitable climate conditions for species (e.g. Peterson, 2001; Pearson *et al.*, 2002; Simmons *et al.*, 2004; Coetzee *et al.*, 2009; Huntley and Barnard, 2012). Bioclimatic envelope models rely on identification of species-specific climate envelopes, inferred from climate conditions experienced within current species' ranges. Such models provide a useful first approximation of species' potential vulnerability to climate change (Pearson and Dawson, 2003) but contain no information on the mechanistic links between species' distributions and climate.

Understanding the mechanistic links between climate space and species persistence matters to predictions of species vulnerability. This is partly because some species may be able to persist in thermal conditions both hotter and colder than the air temperature extremes that currently occur within their range, as well as perhaps under different precipitation regimes. This can occur because a species' current range may not include all areas that are climatically suitable, owing to the presence of other factors (e.g. dispersal barriers or biotic interactions) that limit their distribution (Araújo and Peterson, 2012; Guisan *et al.*, 2014). Additionally, the operative temperature to which an animal is exposed in its environment (the overall thermal environment experienced; Bakken, 1992) depends greatly on habitat use and habitat structure. For example, an animal may experience temperatures much higher than air temperature if foraging in the sun, but may be able to buffer itself behaviourally from such extremes by using micro-refugia (Kearney *et al.*, 2009; Hetem *et al.*, 2011; Cunningham *et al.*, 2015). Importantly, behavioural adjustments may result in costly trade-offs, such as an increased risk of predation, or reduction in energy gain (e.g. Cunningham *et al.*, 2015). Such trade-offs may be unsustainable in the long term (e.g. Sinervo *et al.* 2010) but may not be captured by bioclimatic envelope models that assume species' current distribution is in equilibrium with climate conditions. Knowledge of the physiological and behavioural mechanisms species use to adjust to changing climatic conditions, and the limits of these, can therefore greatly improve the accuracy of predictions of climate change vulnerability (Williams *et al.*, 2008; Araújo and Peterson, 2012; Briscoe *et al.*, 2014). Physiological limits are often considered fundamentally important in predicting species' responses to climate change (Kearney and Porter, 2009; Monahan, 2009; Kearney *et al.*, 2010; Angert *et al.*, 2011; Sunday *et al.*, 2012; Khaliq *et al.*, 2014).

The Fynbos biome of south-western South Africa is a global biodiversity hotspot and a renowned centre of endemism (Myers *et al.*, 2000). Fynbos is the most species-rich of the world's six floral kingdoms (Cowling and Hilton-Taylor, 1994; Cowling *et al.*, 1998; Conservation International, 2014). The region is also home to a rich birdlife, including six endemic passerines (BirdLife International, 2014a). Fynbos vegetation is found only in a small (entirely contained within the borders of a single country), fragmented and mountainous region and is therefore at risk of being heavily affected by climate change, owing to limited capacity for Fynbos species to shift their ranges (Thuiller *et al.*, 2005; Huntley and Barnard, 2012; Foden *et al.*, 2013). Moreover, because it is located within a Mediterranean climate zone, the limited lowland and coastal Fynbos areas are greatly threatened by ongoing fragmentation, because of land development for agriculture and human habitation (Rebelo, 1992; Sala *et al.*, 2000; Hoekstra *et al.*, 2004).

Analyses of long-term weather station data (two studies spanning the periods 1950–2000 and 1961–2009) show significant overall warming trends for the southwest of South Africa, including the Fynbos biome (Warburton *et al.*, 2005; Kruger and Sekele, 2013). This is particularly the case for coastal Fynbos regions, where significant increases in the frequency and magnitude of warm extremes have been accompanied by significant decreases in the frequency and magnitude of cool extremes (Kruger and Sekele, 2013; van Wilgen *et al.*, 2015). The endemic avifauna of the Fynbos has been identified as potentially being highly vulnerable to such warming in bioclimatic envelope modelling exercises (Huntley and Barnard, 2012). However, data on physiological and behavioural responses to temperature are lacking for the six Fynbos-endemic bird species.

In this study, we assessed whether physiological thermal thresholds at high temperatures might underlie changes in range and population size of 12 Fynbos-resident bird species, including the six endemic passerines and six other resident, but non-endemic species. We defined ‘physiological thermal thresholds’ as temperature thresholds for increased costs of physiological thermoregulation in hot conditions, and we also assessed rates of increase in those costs above these thresholds.

First, we followed Jiguet *et al.* (2006, 2010) and determined whether there was a correlation between documented changes in range and population size between two bird atlas data collection periods (1987–1991 and 2007–present; www.sabap2.adu.org.za) and overall mean annual temperatures within each species' range and/or changes in mean annual temperature over the relevant time period. In addition, we assessed whether declines in bird populations as captured by bird atlas data were specifically occurring in areas where warming trends were detected. Second, we aimed to establish whether the patterns we found were supported by the physiological thermal thresholds of each species, perhaps providing evidence of a mechanistic underpinning for the relationship between climate and population trends under climate warming, in cases where significant correlations were found. Specifically, we investigated the following factors: (i) whether species occupying generally cooler ranges showed lower physiological thermal thresholds than species occupying warmer ranges; and (ii) whether physiological thermal thresholds were correlated with observed population trends in warming areas. Given that the Fynbos experienced high climatic stability over evolutionarily relevant temporal scales (Dynesius and Jansson, 2000; Cowling and Lombard, 2002; Linder, 2003; Cowling *et al.*, 2015), we expected Fynbos-endemic species with ranges most restricted to cool regions to show lower thermal thresholds than non-endemic species that experience higher temperatures outside of the biome. In addition, we hypothesized that species vulnerable to warming because of thermal physiological limitations should show declines specifically in areas of their ranges with strong warming trends, and additionally show low thermal thresholds. Finally, we placed our results in a global context, by comparing physiological thermal thresholds of Fynbos birds with those of similar-sized species documented in the literature (McKechnie and Wolf, 2010; Whitfield *et al.*, 2015), particularly species from hotter and more tropical climates.

## Materials and methods

### Study species

We studied the six passerine species endemic to the Fynbos biome and six species that are resident within, but not restricted to, the Fynbos (‘non-endemics’; Table 1, online supplementary material Fig. S1). Non-endemic species were matched to an endemic counterpart (such that six ‘pairs’; were studied), in terms of body size and dietary guild, and taking into account their relative abundances at the study site. Data on biogeography (endemic or non-endemic) were extracted from Hockey *et al.* (2005). Despite recently documented range contractions in all six Fynbos endemics (Lee and Barnard, 2012), all 12 study species are currently listed as Least Concern according to the IUCN Redlist (IUCN, 2015).

**Table 1:** Avian species in which we investigated physiological thermal thresholds at Blue Hill Nature Reserve, South Africa

Pair	Species	Fynbos endemism	Dietary guild	*n*	*M*_b_ (mean ± SEM; g)	MAT (°C)
1	Cape sugarbird (*Promerops cafer*) Linnaeus 1758	E	Nectarivore	11	35.6 ± 1.2	15.5
1	Malachite sunbird (*Nectarinia famosa*) Linnaeus 1766	N	Nectarivore	5	15.1 ± 0.9	15.7
2	Orange-breasted sunbird (*Anthobaphes violacea*) Linnaeus 1766	E	Nectarivore	10	9.4 ± 0.4	15.3
2	Southern double-collared sunbird (*Cinnyris chalybeus*) Linnaeus 1766	N	Nectarivore	10	7.6 ± 0.2	16.4
3	Cape siskin (*Crithagra totta*) Sparrman 1786	E	Granivore	10	12.7 ± 0.3	15.3
3	Cape canary (*Serinus canicollis*) Swainson 1838	N	Granivore	4	15.0 ± 0.4	15.6
4	Protea seedeater (*Crithagra leucoptera*) Sharpe 1871	E	Granivore	9	20.5 ± 0.4	15.0
4	Cape bunting (*Emberiza capensis*) Linnaeus 1766	N	Granivore	10	20.0 ± 0.5	15.8
5	Victorin's warbler (*Cryptillas victorini*) Sundevall 1860	E	Insectivore	6	16.6 ± 0.4	15.2
5	Cape grassbird (*Sphenoeacus afer*) Gmelin 1789	N	Insectivore	5	30.0 ± 1.1	16.2
6	Cape rockjumper (*Chaetops frenatus*) Temminck 1826	E	Insectivore	10	53.6 ± 1.4	14.3
6	Familiar chat (*Cercomela familiaris*) Stephens 1826	N	Insectivore	10	20.5 ± 0.4	16.7

Species have been grouped in endemic and non-endemic pairs (Pair). For each species, status regarding endemism to the Fynbos biome (E = Fynbos endemic; N = non-endemic), dietary guild, sample size (*n*), mean body mass (*M*_b_) at time of capture and mean annual temperature (MAT) of the species' range (Hijmans *et al.*, 2005) are provided (see Materials and methods for details).

### Extraction of data on range and population trends and thermal profiles of ranges

#### Southern African Bird Atlas (SABAP) data

The first and second Southern African Bird Atlas Projects (SABAP1, 1987–1991 and SABAP2, 2007–present) allow comparisons of avian distributions in South Africa ∼15 years apart. Data for these projects consist of species reports submitted by citizen scientists (Harrison *et al.*, 1997). During SABAP1, data collection was conducted at the quarter-degree grid cell (QDGC) level, whereas for SABAP2, data were collected at the pentad level (approximately 9 km × 9 km cells); there are nine pentads in a QDGC. Citizen scientists (birders) submit lists of species observed (seen or heard) within a quarter-degree grid cell (SABAP1) or pentad (SABAP2). Data are collected during an initial 2 h or longer period spent in the grid cell, followed by additional sightings collected over the following 5 days (Péron and Altwegg, 2015). The number of times a species appears on lists submitted for any particular QDGC or pentad is the reporting rate, a metric of relative abundance, likely to be related monotonically to true abundance in the field (Hofmeyr *et al.*, 2014). Data are available for SABAP2 collated to the QDGC level (http://sabap2.adu.org.za/). We therefore compared range changes (species presence or absence between projects) at the scale of QDGCs. We further calculated mean reporting rate changes for each species (which we used as a proxy for population changes) by calculating the mean reporting rate across all QDGCs for each SABAP period. We then calculated the change in mean reporting rate between SABAP1 and SABAP2 as a percentage of the mean SABAP1 reporting rate. Data were accessed from http://sabap2.adu.org.za/ on 27 May 2014.

#### Thermal profiles of species' ranges

The centre of each pentad from which a species had been recorded during SABAP2 was used to extract data on the mean annual temperature (variable name ‘Bio 1’) of that point from the Worldclim database (resolution ∼1 km; Hijmans *et al.*, 2005). For each species, we calculated the mean of the Bio 1 values (i.e. average of mean annual temperature, hereafter ‘MAT’) from across the species' entire range (Table 1).

#### Climate change within species' ranges

The Worldclim database provides high-resolution data based on a multiyear average (1950–2000). In order to assess change in climate within our study species' ranges, we used coarser-scale data (half-degree grid cell resolution) from the University of Delaware's Gridded Monthly Data Set (version 3.01; Matsuura and Willmott, 2012). We extracted data on temperature (mean annual temperature; in degrees Celsius) for each year within SABAP1 (1987–1991) and SABAP2 (2007–2010; more recent data were unavailable). Although changes in maximal temperature could be biologically more meaningful, the University of Delaware's Gridded Monthly Data Set's finest temporal resolution is monthly means. Furthermore, weather station densities in the study region are too low to be of use in extracting historical data on maximal temperatures. We therefore matched mean annual temperature data to the quarter-degree grid cell scale of the SABAP data by assigning the same temperature measure to each of the four QDGCs contained within each half-degree grid cell. For each QDGC, we then calculated the average of the mean annual temperatures across all the years included in SABAP1 and SABAP2, respectively. The magnitude and direction of change in temperature between SABAP1 and SABAP2 was calculated by subtracting mean SABAP1 temperature from mean SABAP2 temperature. Previous work has verified that such comparison reflects linear rates of temperature change within QDGCs (Madden, 2013).

### Collection of data on physiological thermal thresholds

#### Study site

We collected physiological data on 12 passerine bird species (Table 1) from 23 September to 29 November 2013 (late spring to early summer) at Blue Hill Nature Reserve (33.59S; 23.41E; 2230 ha; 1000–1530 m above sea level), Western Cape, South Africa. Blue Hill Nature Reserve has mountainous topography, with Mountain Fynbos as the dominant vegetation type (Lee and Barnard, 2013; Mucina and Rutherford, 2006). It lies in a transition zone between summer and winter rainfall regions; mean annual rainfall is 397 ± 98 mm (Lee and Barnard, 2013). Mean daily temperature (24 h period) recorded by an onsite weather station (Davis Vantage Vue, USA) during the study period was 15.0°C (range, 6.6–24.5°C), with maximal and minimal temperatures of 33.5 and −1.6°C, respectively.

#### Collection of physiological data

We measured the temperature dependence of evaporative water loss (EWL), resting metabolic rate (RMR) and body temperature (*T*_b_) in all 12 species. All birds were captured between dawn and midday (during the active phase of their circadian cycles) using mist nets or spring traps. After capture, each bird was weighed (accurate to 0.1 g) and ringed with a uniquely numbered aluminium band for identification. We used only non-breeding adult birds in the study; all others were released after ringing. Age and sex (where possible) were determined from plumage, and breeding status was assessed by examining brood patches and looking for smear marks from provisioning on beaks.

Physiological measurements were carried out using an open-flow field respirometry system set up in a building on the study site (details of procedures are provided in the online supplementary material). Measurements included EWL, RMR [measured as rate of carbon dioxide production (${\dot{V}}_{CO_{2}}$) and oxygen consumption (${\dot{V}}_{O_{2}}$)] and *T*_b_, obtained at air temperatures (*T*_air_) ranging from 24 to 42°C. Each individual was exposed to a maximum of five test temperatures (ramped profile), and the total duration of each experimental run was <4 h (see online supplementary material).

### Data analyses

All analyses were carried out in the R statistical environment (R Development Core Team, 2013). In all cases, values of *P* < 0.05 are indicative of significance.

#### Correlations between SABAP data and temperature data

For each species, we calculated Pearson's correlation coefficients for the relationships between MAT and temperature change with species' ranges, as well as range changes and abundance changes (changes in reporting rate) between SABAP1 and SABAP2. We also split species' abundance change data into QDGCs in which they showed declines or extinction and QDGCs in which they were stable or increased between SABAP1 and SABAP2. We used Walsch two-sample *t*-tests (two tailed) to assess the strength and direction of temperature change within these two groups of cells.

Finally, in order to assess whether Fynbos endemics occupied cooler ranges than non-endemics, we compared mean MAT for Fynbos endemics vs. non-endemics using a Walsch two-sample *t*-test (two tailed).

#### Physiological data

Davies' tests using the *segmented* package in R (Muggeo, 2003) were used to detect changes in the slopes of relationships between *T*_air_ and EWL, RMR or *T*_b_. Where the Davies' test identified a significant change in a slope, we used broken stick regression analyses in the *segmented* package (Muggeo, 2003) to estimate the inflection points in *T*_air_ (‘thermal thresholds’) above which EWL, RMR and *T*_b_ started to increase for each species (inflection *T*_air_ of EWL, RMR and *T*_b_ were defined as *T*_ewl_, *T*_uc_ and *T*_tb_, respectively). Rate of change (slope) in each of these variables above the inflection *T*_air_ was assessed using linear mixed-effects regression models (with individual as random factor) in the *nlme* package in R (Pinheiro *et al.*, 2013). We further calculated the relative increase in EWL (EWL-change) and *T*_b_ (*T*_b_-change) shown by each species between *T*_air_ at 30°C (likely to fall within the thermoneutral zone of most species; Khaliq *et al.*, 2014) and 38°C (the warmest test *T*_air_ shared by all species during the study). For EWL, we calculated the mean ratio of EWL measurements at *T*_air_ = 38°C to EWL measurements at 30°C, whereas for *T*_b_ we calculated mean increase in *T*_b_ (i.e. *T*_b_ at 38°C minus *T*_b_ at 30°C). The latter two metrics allowed us to estimate the relative magnitude of thermoregulatory cost that species are likely to experience at a high *T*_air_. All variables were calculated as mass specific (i.e. per gram body mass).

#### Interspecific comparison of Blue Hill Nature Reserve species

We tested whether any of the above-listed EWL, RMR and *T*_b_ responses varied with the following parameters: (i) body mass (*M*_b_); and (ii) endemism and MAT.

Body mass generally has a large effect on physiological variables (Tieleman and Williams, 2000; McKechnie and Wolf, 2004, 2010). To account for this, we also determined the size of the effect of body mass on mass-specific physiological variables through least-squares regression analyses and extracted partial residuals. We then used these residuals to test for an effect of MAT and endemism, and the two-way interaction between these two factors, once the effect of body mass was removed.

Cape rockjumper (*Chaetops frenatus*) was an outlier in this data set in terms of both MAT (very cool) and *M*_b_ (larger than other species tested). We therefore present analyses both including and excluding Cape rockjumper, in order to assess the degree to which this species was driving the results.

The small sample size in terms of number of species in the above analyses precluded us from conducting additional phylogenetic independent analyses (Blomberg *et al.*, 2003).

#### Global multispecies comparison

McKechnie and Wolf (2010) determined allometric relationships of *T*_ewl_ and the slope of the EWL relationship above *T*_ewl_ (drawn from the global literature for 27 bird species) and showed that both traits were negatively correlated with body size (*r*^2^ for EWL slope = 0.859 and *r*^2^ for *T*_ewl_ = 0.487). In order to understand thermal physiological responses of Fynbos birds in a global context, we performed both conventional generalized least-squares regression analyses and phylogenetic least-squares (PGLS) analyses to determine how allometric scaling of *T*_ewl_ and EWL slope vary at a global scale (see online supplementary material). We included *T*_ewl_ and EWL slope data for species measured in the present study, species presented by McKechnie and Wolf (2010) and an additional five South African species (Whitfield *et al.*, 2015). We limited the comparative analyses to species weighing <90 g, because data on larger species are scant and this smaller range better represents the *M*_b_ values of the species we studied. We compared species according to the world climatic zone in which most of their distribution is centred, as classified by W. Köppen in 1900 (updated version: Kottek *et al.*, 2006). Categories included tropical, desert and temperate (we use temperate for simplicity, although most of the Fynbos endemics have a Mediterranean distribution). Three species, malachite sunbird (*Nectarinia famosa*), familiar chat (*Cercomela familiaris*) and common nighthawk (*Chordelius minor*), had distributions stretching across two or more climate zones. Upon visual inspection of the data, malachite sunbird and familiar chat (both measured in the present study) were similar to other Fynbos species and were therefore included in the temperate group. Common nighthawk was grouped with tropical birds; given that this species is migratory and overwinters mainly at tropical latitudes (BirdLife International, 2014b), it will generally experience warm weather year-round.

#### Model selection and presentation

We used Akaike Information Criteria scores adjusted for small sample sizes (AIC_c_) to select the best-fitting models for each physiological parameter in the global multispecies comparison. Where AIC_c_ values were similar (ΔAIC_c_ < 2) and models were nested, the simplest model with the least explanatory factors was selected as the best model, following Arnold (2010). All data are either mass specific or are partial residuals of linear regressions on *M*_b_, as indicated, and are presented as means ± SEM per individuals measured (*n*) for each species.

## Results

### Relationships between temperature and SABAP reporting rate and range changes

**Figure 1:**
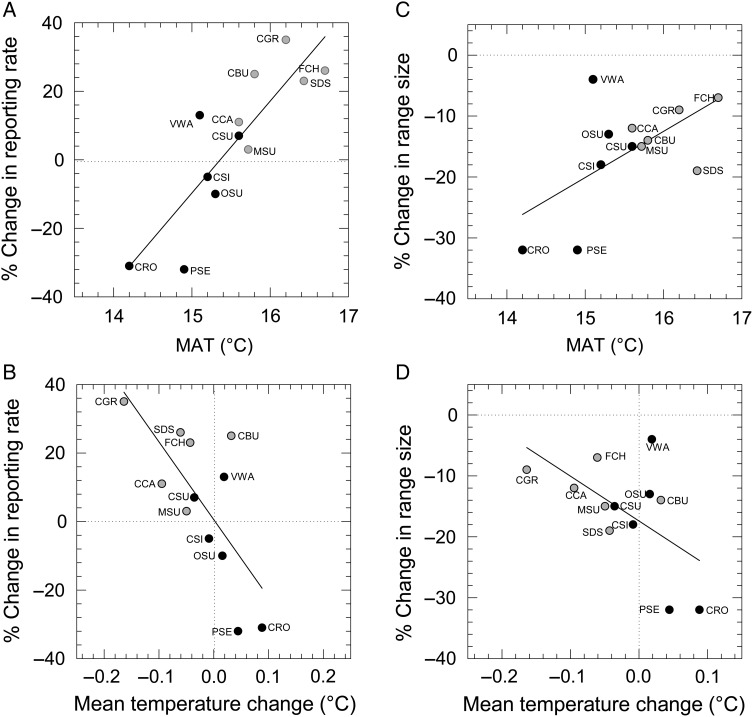
(**A**,**B**) There was a significant correlation between change in reporting rates of Fynbos resident bird species between SABAP1 and SABAP2 and mean annual temperature of each species' range (MAT, A). In addition, there was also a significant correlation between changes in reporting rates and change in temperature within each species' range between SABAP1 and SABAP2 (B). (**C**,**D**) There were also significant correlations between the degree of range contraction of Fynbos resident bird species between SABAP1 and SABAP2 and mean annual temperature of each species' range (MAT; C), and change in temperature within each species' range between the two survey periods (D). Black circles represent Fynbos endemics and grey circles represent non-endemics. Each species' data point is labelled with a three-letter acronym, as follows: CBU, Cape bunting; CCA, Cape canary; CGR, Cape grassbird; CRO, Cape rockjumper; CSI, Cape siskin; CSU, Cape sugarbird; FCH, familiar chat; MSU, malachite sunbird; OSU, Orange-breasted sunbird; PSE, Protea seedeater; SDS, Southern double-collared sunbird; VWA, Victorin's warbler.

Despite variability in the data, there was a significant positive correlation between MAT and change in species reporting rate (proxy for abundance) between SABAP1 and SABAP2 (Pearson's correlation coefficient, *r* = 0.86, *t*_1,10_ = 5.23, *P* < 0.001) such that warmest-MAT species in general fared better (reporting rates increased) than species with coolest MAT (declining reporting rates; Fig. 1A). Likewise, there was a significant negative relationship between change in mean annual temperature between SABAP1 and SABAP2 within a species' range and change in species reporting rate such that species occupying ranges that warmed the most between the two periods [e.g. Cape rockjumper and Protea seedeater (*Crithagra leucoptera*)] experienced declines, whereas species with cooling ranges tended to increase [e.g. Cape Canary (*Serinus canicollis*) and Cape grassbird (*Sphenoeacus afer*); *r* = −0.71; *t*_1,10_ = −3.22, *P* = 0.009; Fig. 1B].

All 12 of our study species show reductions in range size between SABAP1 and SABAP2. There was a significant positive correlation between the amount of range contraction and MAT (*r* = 0.60, *t*_1,10_ = 2.38, *P* = 0.04), such that species with warmer MAT in general experienced smaller range contractions (Fig. 1C). In addition, there was a significant negative relationship between change in mean annual temperature between SABAP1 and SABAP2 within a species' range and change in range size for that species, such that species occupying ranges that warmed most between the two periods generally experienced the largest range contractions, whereas species with stable or cooling ranges experienced the smallest range contractions (*r* = −0.58, *t*_1,10_ = −2.23, *P* = 0.05, Fig. 1D). Welch two-sample *t*-tests confirmed that Fynbos endemics occupy significantly cooler ranges than non-endemics (mean MAT ± 1 SEM: for Fynbos endemics, 15.1 ± 0.2°C; for non-endemics, 16.1 ± 1.2°C; *t*_1,10_ = −3.88, *P* = 0.003) and have experienced larger warming trends (mean change in MAT between SABAP1 and SABAP2 ± 1 SEM: for Fynbos endemics, 0.02 ± 0.02°C; for non-endemics, −0.06 ± −0.03°C; *t*_1,10_ = 2.65, *P* = 0.027).

University of Delaware climate data (Matsuura and Willmott, 2012) showed that warming had not been uniform across the Fynbos biome between SABAP1 and SABAP2. Highest rates of warming had occurred in inland (mountainous) areas, and some coastal areas had experienced cooling (online supplementary material Fig. S2). Eight of our 12 study species (and five of six Fynbos endemics) showed declines in reporting rate or local extinction in >50% (more than half of the quarter-degree grid cells) of their combined SABAP1 and SABAP2 ranges. However, only two of these species [Cape rockjumper and Cape bunting (*Emberiza capensis*)] experienced statistically significant overall warming across their ranges.

Of these two species, reporting rates of Cape buntings increased between SABAP1 and SABAP2, and this was associated with warming (areas with increasing Cape bunting populations also showed significant warming trends, whereas areas with declines had no significant temperature trend). The Cape rockjumper showed the opposite pattern, with declines in >70% of its range and an overall decline in reporting rate. Furthermore, areas in which rockjumpers declined showed significant warming trends, whereas areas where the population was stable or increased showed no significant temperature trend.

Victorin's Warbler (*Cryptillas victorini*) likewise showed declines only in areas of its range with significant warming trends. However, the range of this species did not warm significantly overall between SABAP1 and SABAP2, and neither does the bird appear to be in decline overall. Protea seedeater showed a similar magnitude of overall decline to Cape rockjumper. Like the Cape rockjumper, this species had cool MAT and a significant warming trend across its range. However, Protea seedeater reporting rate declines did not occur specifically in warming areas of its range (Table 2).

**Table 2:** Temperature and population changes within the ranges of 12 Fynbos-resident bird species

Species	Fynbos endemism	Mean temperature change (°C) population increases	Mean temperature change (°C) population decreases	Mean temperature change (°C) overall	Percentage of QDGCs within range with declines	Reporting rate change (%)
Cape rockjumper	E	0.02	0.11**	0.09***	71	−31
Victorin's warbler	E	−0.04	0.10*	0.02	41	13
Protea seedeater	E	0.07	0.04	0.04	72	−32
Orange-breasted sunbird	E	0.01	0.02	0.016	62	−10
Cape sugarbird	E	−0.05	−0.03	−0.04	61	7
Cape siskin	E	0.05	−0.05	−0.01	61	−5
Cape grassbird	N	−0.17***	−0.16***	−0.16***	37	35
Southern double-collared sunbird	N	−0.05	−0.03	−0.04*	50	23
Cape canary	N	−0.09***	−0.10***	−0.09***	53	11
Malachite sunbird	N	−0.03	−0.07***	−0.05***	58	3
Cape bunting	N	0.08***	−0.02	0.03*	51	25
Familiar chat	N	−0.04*	−0.09***	−0.06***	43	26

Abbreviations: E, Fynbos endemic; N, Fynbos non-endemic. Reporting rate change provides information on overall abundance changes of each species between SABAP1 and SABAP2 (positive values indicate increases in abundance; negative values indicate declines). QDGCs is the quarter-degree grid cells, i.e. the resolution at which SABAP1 data were collected. Temperature change data are presented separately for QDGCs in which each species showed abundance declines or extinctions (decreases); QDGCs in which the species was stable or increased in abundance (increases); and overall (across the entire species' range). Note that Cape rockjumper experienced a reduction in reporting rate, declined in >70% of its range between SABAP1 and SABAP2 and showed a significant warming trend across the range, driven by warming specifically in areas where it had declined in abundance. By comparison, the Protea seedeater also experienced a reduction in reporting rate and showed declines in >70% of its range, but there was no evidence of climate warming playing a role. *P < 0.05; **P < 0.01; ***P < 0.001.

### Blue Hill Nature Reserve physiological data

#### Mass-specific evaporative water loss

Davies' test identified significant upper inflection points for EWL as a function of *T*_air_ (*T*_ewl_) for 10 of the 12 study species (online supplementary material Table S1 and Fig. S3). Owing to limited sample sizes at high *T*_air_, we excluded Cape canary from *T*_ewl_ and slope analyses. Davies' test identified a near-significant *T*_ewl_ of 34.7°C in Victorin's warbler (*P* = 0.09). We included this species in analyses of *T*_ewl_ because excluding it did not affect the outcome of these analyses. The *T*_ewl_ ranged from 31.3°C in Cape rockjumper to 37.5°C in Southern double-collared sunbird (*Nectarinia chalybea*; overall mean = 34.6°C). All study species show panting behaviour above their respective *T*_ewl_ value (online supplementary material Table S2).

**Figure 2:**
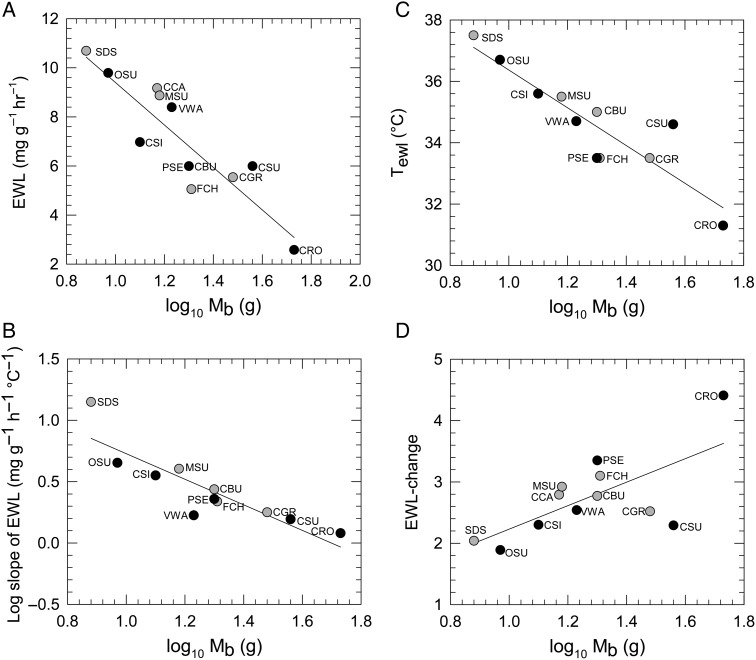
Evaporative water loss (EWL, approximating levels within the thermoneutral zone; **A**), rates of increase in EWL above inflection air temperature for EWL (log_10_ slope of EWL; **B**), inflection air temperatures for EWL (*T*_ewl_; **C**) and EWL-change (ratio of EWL at *T*_air_ = 38°C to EWL at *T*_air_ = 30°C; **D**) were significantly related to log_10_ body mass (log_10_*M*_b_). Black circles represent Fynbos endemics and grey circles represent non-endemics. Trend lines represent significant relationships (*P* < 0.05). Each species' data point is labelled with a three-letter acronym, as in Fig. 1.

All evaporative cooling responses were significantly related to log_10_*M*_b_ as follows: EWL at 30°C (negatively, *F*_1,10_, = 37.64, *P* < 0.001); log_10_ EWL slope (negatively, *F*_1,8_ = 30.35, *P* < 0.001); *T*_ewl_ (negatively, *F*_1,9_ = 37.44, *P* < 0.001); and EWL-change (positively, *F*_1,10_ = 8.640, *P* = 0.015; Fig. 2).

#### Mass-corrected residual evaporative water loss

**Figure 3:**
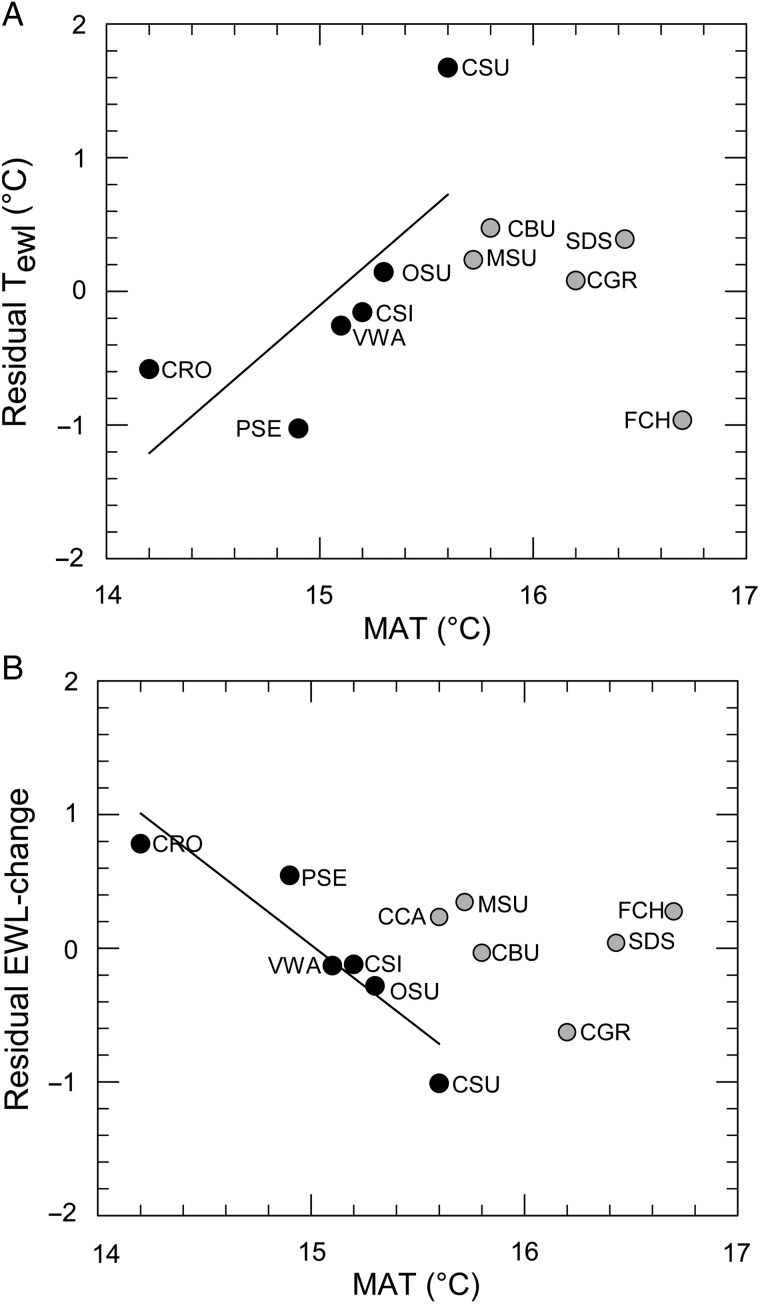
The relationship between mean annual air temperature (MAT; in degrees Celsius) experienced across each species' range, and mass-corrected residuals of upper inflection air temperature for evaporative water loss (*T*_ewl_; **A**) and EWL-change (ratio of EWL at *T*_air_ = 38°C to *T*_air_ at 30°C; **B**). Black circles represent Fynbos endemics and grey circles represent non-endemics. Trend lines represent significant relationships (*P* < 0.05; see main text for details on interaction effects). Each species' data point is labelled with a three-letter acronym, as in Fig. 1.

Residual EWL at 30°C and residual slope of EWL did not vary with endemism, MAT or interaction effects (all *P*-values '> 0.300); however, there was a marginally significant interaction effect of endemism and MAT (*F*_1,7_ = 6.075, *P* = 0.0432) on residual *T*_ewl_. When we analysed data for Fynbos endemics and non-endemics separately, residual *T*_ewl_ showed a positive, non-significant correlation with MAT (*F*_1,4_ = 4.060, *P* = 0.114) for endemic birds, but non-endemic birds showed a negative, non-significant correlation (*F*_1,3_ = 3.08 = 0.178; Fig. 3A). However, after removing Cape rockjumper from these analyses, the interaction effect of MAT and endemism on *T*_ewl_ became much stronger (*F*_1,6_ = 32.76, *P* = 0.001), with Fynbos endemics showing a significant positive correlation of *T*_ewl_ with MAT (*F*_1,3_ = 86.74, *P* = 0.003). Residual EWL-change was affected by an interaction between MAT and endemism (*F*_1,8_ = 5.456, *P* = 0.048; Fig. 3B). Residual EWL-change was on average greater in Fynbos endemics than in non-endemics, and had a negative relationship with MAT. After removing Cape rockjumper from the analyses, the interaction between MAT and endemism became more significant (*F*_1,7_ = 8.552, *P* = 0.022). Residual EWL-change showed a significant negative relationship to MAT in Fynbos endemics (with Cape rockjumper, *F*_1,4_ = 21.904, *P* = 0.009; without Cape rockjumper, *F*_1,3_ = 74.768, *P* = 0.003). In contrast, non-endemics showed no relationship between residual EWL-change and MAT (*F*_1,4_ = 0.090, *P* = 0.779; Fig. 3B).

#### Mass-specific resting metabolic rate

Intraspecific variation in RMR values was generally high. Most of the 12 species showed negative relationships between RMR and *T*_air_. Despite this, in most species, individuals' RMR was slightly higher at high *T*_air_ (40°C). However, with the exception of familiar chat, we could identify only lower, but not upper, inflection points of the thermal neutral zone, probably because of limited exposure at higher *T*_air_ (online supplementary material, Fig. S4 and Table S3). At an interspecific level, there was a significant negative relationship between RMR at *T*_air_ = 30°C and log_10_*M*_b_ (*F*_1,9_ = 5.952, *P* = 0.037); the largest species, Cape rockjumper, showed the lowest RMR at *T*_air_ = 30°C (36.73 J g^−1^ h^−1^), and the small sunbirds, Cape siskin (*Serinus totta*) and Protea seedeater, showed RMR > 70 J g^−1^ h^−1^ at *T*_air_ = 30°C (online supplementary material, Table S4). Residual RMR did not vary with MAT or endemism or the interaction effect between these (*F*_3,7_ = 0.1782, *P* = 0.908).

#### Body temperature

**Figure 4:**
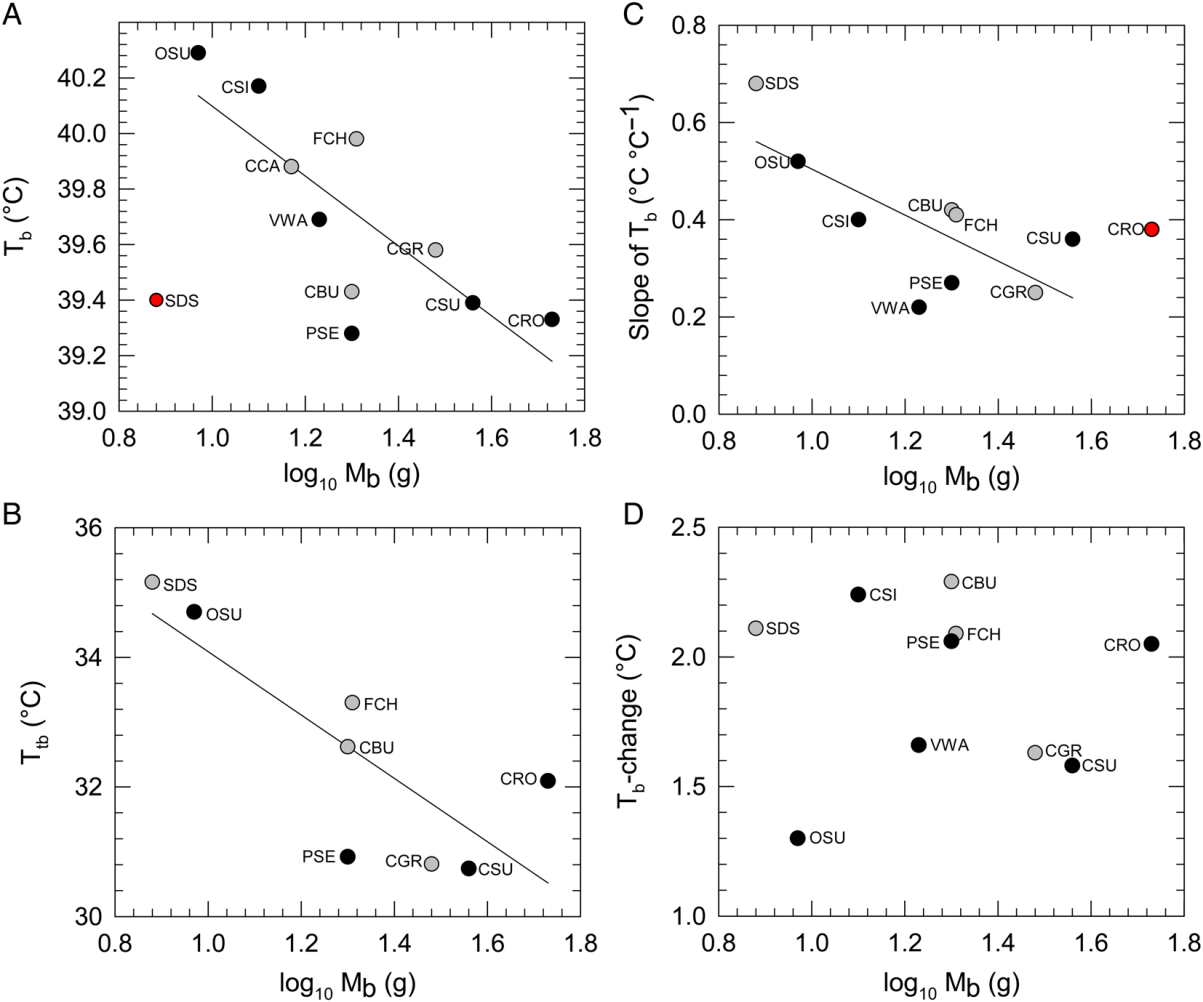
Body temperature (*T*_b_, approximating levels within the thermoneutral zone; **A**), inflection air temperature for *T*_b_ (*T*_tb_; **B**), rates of increase in *T*_b_ above inflection air temperature for *T*_b_ (slope of *T*_b_; **C**) and *T*_b_-change (*T*_b_ at *T*_air_ = 38°C minus *T*_b_ at *T*_air_ = 30°C; **D**) as a function of log_10_ body mass (log_10_*M*_b_). Black circles represent Fynbos endemics and grey circles represent non-endemics. Trend lines represent significant relationships (*P* < 0.05), excluding outliers (red circles). Each species' data point is labelled with a three-letter acronym, as in Fig. 1.

Body temperature showed a negative correlation with log_10_*M*_b_. Southern double-collared sunbird was an outlier (lower *T*_b_ than expected for its size) and upon removal of this datum from the analyses, *T*_b_ showed a significant negative relationship with log_10_*M*_b_ (*F*_1,8_ = 12.48, *P* = 0.008; Fig. 4A). With the exception of Cape canary, all species showed elevations in *T*_b_ at high *T*_air_ (online supplementary material, Table S5 and Fig. S5). Significant upper inflection *T*_air_ values for *T*_b_ (*T*_tb_) were identified in only eight species and ranged from *T*_air_ = 30.8°C in Cape grassbird (*Sphenoeacus afer*) to *T*_air_ = 35.2°C in Southern double-collared sunbird (mean ± SD = 32.1 ± 2.33°C; Fig. 4B). The *T*_tb_ varied negatively with log_10_*M*_b_ (*F*_1,6_ = 10.75, *P* = 0.017) but was not affected by MAT or endemism (all *P*-values > 0.5).

Mean slope of *T*_b_ above *T*_tb_ was 0.39 ± 0.14°C *T*_b_°C^−1^*T*_air_ (range 0.22 in Victorin's warbler to 0.68 in Southern double-collared sunbird) and showed a near-significant, negative correlation with log_10_*M*_b_ (*F*_1,8_ = 4.512, *P* = 0.066). The slope of *T*_b_ increase in Cape rockjumper appeared high relative to its *M*_b_ (0.38°C *T*_b_°C^−1^*T*_air_), and upon removal of this species from the analyses, the negative relationship between slope of *T*_b_ increase and log_10_*M*_b_ became significant (*F*_1,7_ = 7.897, *P* = 0.0261), such that smaller species experienced more rapid rates of *T*_b_ increase at high *T*_air_ (Fig. 4C).

Mass-corrected residuals of *T*_b_ slope did not vary with endemism or MAT (all *P*-values > 0.300). There was no effect of log_10_*M*_b_, endemism or MAT (all *P*-values > 0.700) on *T*_b_-change (between *T*_air_ = 30 and 38°C; Fig. 4D). The mean value of *T*_b_-change was 1.9 ± 0.33°C.

### Global multispecies comparison

**Figure 5:**
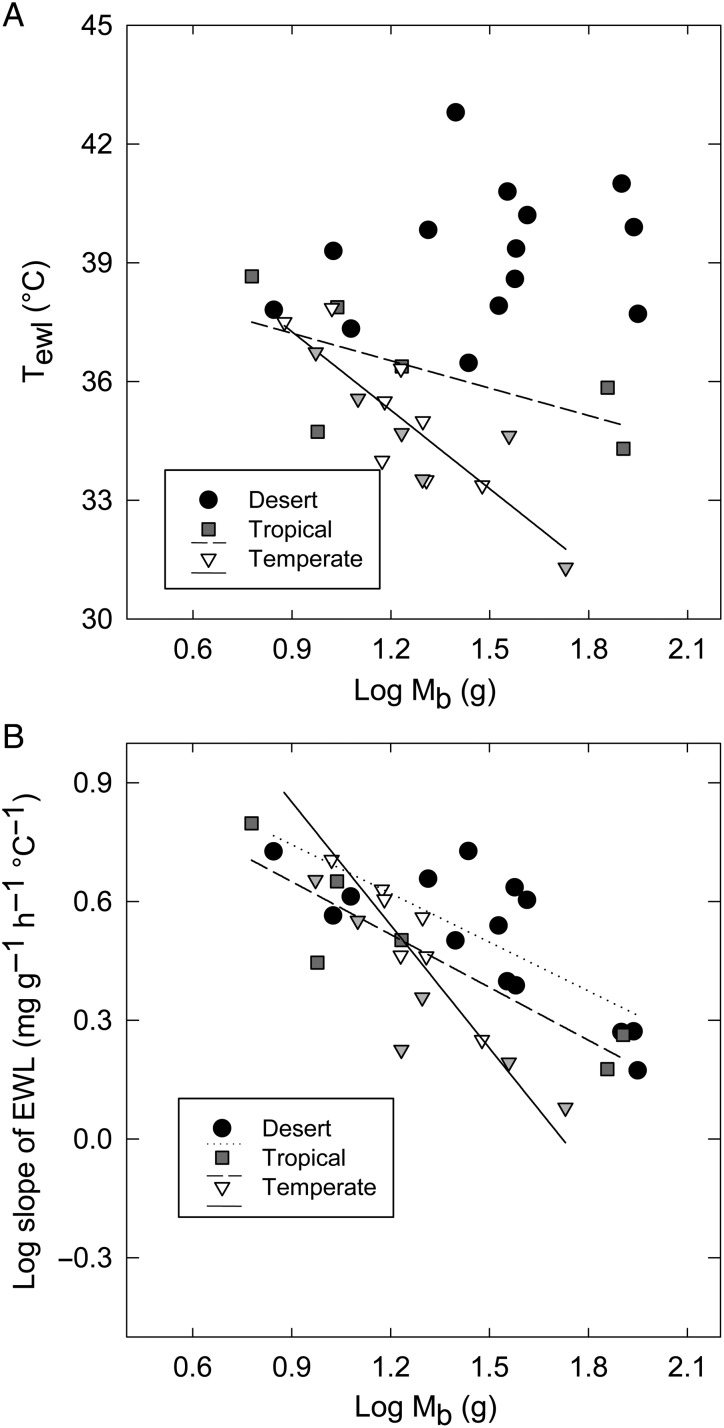
(**A**) The relationship between body mass (*M*_b_; in grams) and mean EWL inflection point (*T*_ewl_; in degrees Celsius), in species from different climate zones. (**B**) Slope of EWL above *T*_ewl_ (EWL slope; in milligrams per gram per hour per degree Celsius) as a function of body mass (*M*_b_; in grams) in species from all three climatic zones. Statistics on conventional and phylogenetic least-squares analyses are presented in the main text. Trend lines represent significant relationships. Symbols represent the following: desert species are represented by black circles, tropical species by grey squares, and temperate species by triangles. Fynbos endemics within the temperate group are illustrated by the grey triangles.

Across the 34 species included in our global multispecies comparison (online supplementary material, Table S6), *T*_ewl_ and EWL slope depended on an interaction between log_10_*M*_b_ and climate zone in both conventional (*T*_ewl_, *F*_5,29_ = 18.2, *P* < 0.001; and EWL slope, *F*_5,29_ = 18.7, *P* < 0.001) and PGLS analyses (*T*_ewl_, *F*_6,29_ = 11.1, *P* < 0.001; and EWL slope, *F*_6,29_ = 12.0, *P* < 0.001; online supplementary material, Table S8). In desert species, *T*_ewl_ showed a weak negative relationship to log_10_*M*_b_ in conventional analyses (*P* = 0.03), but was not significantly related in PGLS analyses (*P* = 0.151; online supplementary material, Tables S7 and S8). In temperate and tropical species, *T*_ewl_ showed a significant negative relationship to log_10_*M*_b_ in both conventional and PGLS analyses (all *P* < 0.01; online supplementary material, Table S8). Consequently, *T*_ewl_ was significantly higher in the larger desert-dwelling species (>15 g) compared with species from other climatic zones and was lowest in the temperate (mostly Fynbos) species (Fig. 5A; online supplementary material, Table S8). For example, Cape rockjumper showed *T*_ewl_ ∼9°C lower than other similar-sized species for which data were available (Fig. 5A). In both conventional and PGLS analyses, EWL slope as a function of log_10_*M*_b_ was significantly negative in birds from all climate zones, but larger temperate birds had significantly lower EWL slopes (*P* < 0.01) than species generally associated with warmer climates (Fig. 5B; online supplementary material, Table S8).

## Discussion

This study provides the first evidence that declines in some Fynbos passerines, notably Cape rockjumper, may be linked to climate warming via low physiological thermal thresholds, whereas evidence is more equivocal for other species. Among Fynbos endemics, climate-change-correlated declines were greater in cool-climate species than in warmer-climate species. In general, this pattern was reflected physiologically only by higher costs of evaporative cooling in cool-climate species, and not by RMR or body temperature trends. Furthermore, we did not find strong evidence of higher thermal thresholds in warmer-climate species resident within, but not endemic to, the Fynbos, despite their notably more positive population trends. This suggests that, with the possible exception of Cape rockjumper, physiological thermal thresholds are unlikely to be the only drivers of most Fynbos bird population changes under recent warming, and other factors that co-vary with climate change may be at least partly responsible.

### Climate, population trends and physiology in the Fynbos passerine community

Population trends of our 12 Fynbos study species were positively correlated with mean annual temperature in species' ranges, so that species occupying coolest ranges declined in reporting rate between 1991 (the completion of SABAP1) and the present (2014), but species occupying the warmest ranges increased. Climate warming has been non-uniform within South Africa over the past few decades (Kruger and Sekele, 2013), and population trends were also negatively correlated with rates of warming, such that species experiencing greatest warming within their ranges declined most. Although average warming within the ranges of Fynbos birds was small (at most in the region of 0.1°C, present study), warming mean temperatures are linked to increased frequency and intensity of high-temperature extremes (van Wilgen *et al.*, 2015), and possibly drive resource bottlenecks, both of which are likely to carry high biological significance (Moreno and Møller, 2011; Cunningham *et al.*, 2013; Maron *et al.*, 2015). In general, Fynbos endemics occupied cooler, but more rapidly warming, ranges than resident non-endemics. In keeping with this, endemics appear to have experienced greater negative impacts of recent climate change (larger reporting rate declines between the two SABAP periods, 1987–1991 and 2007–2014).

Detailed assessments of organisms' responses to climate change are rarer from the southern than the northern hemisphere (Barnard and Thuiller, 2008). However, our findings reflected those of Jiguet *et al.* (2006, 2010) for north temperate birds, and suggested that cool-climate birds globally might show a ‘syndrome’ of vulnerability to climate change, with declines linked to both the historical temperature regime within species' ranges and to rates of warming. Should these correlations have a physiological causal basis, we would expect lower physiological thermal tolerance in cool-climate compared with warmer-climate birds; a pattern that might have evolved through selection for cold tolerance at the expense of heat tolerance (e.g. via low thermal conductance; MacArthur and Wang, 1973). In order to assess physiological thermal tolerance, we looked for thresholds in birds' responses to rising ambient temperature, in terms of *T*_b_, RMR and EWL. Other authors have used lethal thermal limits (e.g. Monahan, 2009) or ‘thermal end points’, above which thermoregulation fails (Whitfield *et al.*, 2015) to assess climate vulnerability, but our protocol was not set up to test these. Moreover, the behavioural changes made by organisms in order to avoid operative temperatures above thermal thresholds can carry considerable costs of their own (e.g. lost foraging opportunities; Sinervo *et al.*, 2010; du Plessis *et al.*, 2012; Cunningham *et al.*, 2015). These have fitness consequences that can be severe enough to threaten population persistence (Sinervo *et al.*, 2010). Therefore, exposure to operative temperatures that elicit costly physiological and behavioural responses, e.g. above the upper critical limit of the thermoneutral zone (*T*_uc_), may impose limits on populations even before lethal temperatures are reached (e.g. Khaliq *et al.*, 2014). Operative temperatures close to *T*_uc_ are also associated with a rapid increase in EWL. This EWL threshold (*T*_ewl_) carries implications for water balance, with attendant risks of dehydration as operative temperatures increase (McKechnie and Wolf, 2010). The *T*_ewl_, and increase in EWL above *T*_ewl_ (EWL-change), may therefore also represent biologically meaningful thermal parameters of relevance to the fitness of organisms.

We found evidence of increased costs of evaporative cooling in cool-climate Fynbos-endemic birds (*T*_ewl_ and EWL-change), but no evidence of similar patterns in non-endemic Fynbos residents. For example, among Fynbos endemics, but not non-endemics, species with historically the coolest ranges tended to have lowest *T*_ewl_. Body temperature and EWL increased significantly at high temperatures in the majority of our study species (endemic and non-endemic), but cool-climate Fynbos endemics are likely to experience greater EWL costs on hot days (days between 30 and 40°C) because of their lower *T*_ewl_ thresholds than non-endemic residents. In contrast, the temperature dependence of RMR showed large intraspecific variation, and we could not identify *T*_uc_ in most species. For each individual bird, the RMR values obtained at air temperatures above 35°C were generally higher, but we suspect that we would need to obtain data at a higher range of *T*_air_ to determine statistically the upper inflection points for RMR. After controlling for body mass effects, the significant correlations of *T*_ewl_ and of EWL-change with MAT (mean annual temperature within species' ranges) suggest that evaporative thermoregulatory demands in Fynbos endemics have become specialized to occupying the cooler climates experienced in their small range. Increasing evaporative cooling costs, especially during high-temperature extremes in summer, may partly explain the apparently greater vulnerability of cool-climate Fynbos endemics to warming trends, which we observed in the SABAP data. Precise mechanisms may include behavioural trade-offs in which birds seek cool microsites in order to avoid rising water costs, but at the expense of foraging opportunities (e.g. Cunningham *et al.*, 2015). It seems likely that the EWL relationships we observed in Fynbos endemics were not seen in non-endemics that occupy larger ranges outside of the Fynbos biome, because they probably experience a wider range of climatic conditions. Species occupying a wider range of habitats and climatic conditions might be able to match their physiological thermal tolerances better to local conditions to minimize costs of thermoregulation (Smit *et al.*, 2013).

### A closer look at individual species

**Figure 6:**
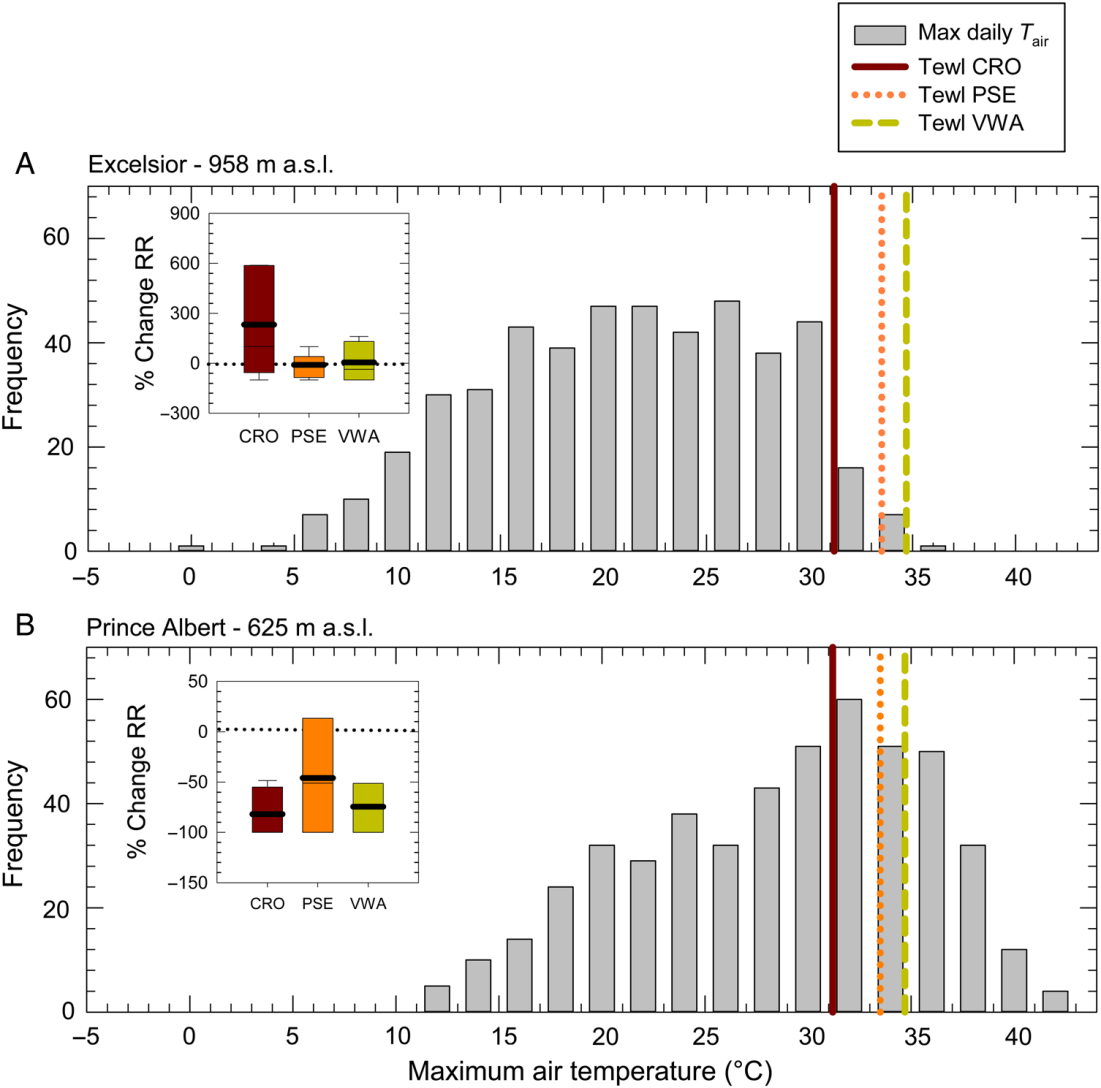
Frequency distribution of maximal daily air temperature (grey bars) recorded between 2013 and 2015 at two inland weather stations within the Fynbos biome: (**A**) Excelsior in foothills of the Cederberg Mountain Range; and (**B**) Prince Albert at the foothills of the Great Swartberg Mountain range. Insets show the mean percentage change in reporting rate of three Fynbos-endemic birds [Cape rockjumper (CRO), Protea seedeater (PSE) and Victorin's warbler (VWA)] observed between SABP1 and SABAP2 in quarter-degree grid cells that border the respective weather stations. Coloured vertical lines represent the air temperature threshold for evaporative water loss (*T*_ewl_) in the above bird species. In warmer regions of the Fynbos, endemic species are more likely to experience *T*_air_ above the *T*_ewl_, and also show significant reductions in reporting rates.

Although correlations between mean annual temperatures in species' ranges (MAT), degree of warming and population trends were strong and significant, there was considerable variation in the data. Closer examination of data from individual species showed large interspecific differences in their thermal physiology and responses to warming. Two species showing population declines (Cape rockjumper and Protea seedeater) and two showing increases (Victorin's warbler and Cape bunting) illustrate these differences particularly well. The Fynbos-endemic species, Cape rockjumper and Protea seedeater, occupy the coolest climatic niches of all our study species and have shown the greatest reductions in relative abundance and range size over the past two decades (overall reporting rate reductions of 31 and 32%, respectively). Declines in Cape rockjumper reporting rates were specifically linked to areas of the species' range with significant warming trends between SABAP1 and SABAP2 (>0.1°C on average), whereas reporting rates were stable or increasing in areas with stable temperatures. However, declines in Protea seedeater reporting rate were not specifically linked to warming areas within the species' range, suggesting that drivers of decline in these two species are likely to differ. The divergent responses of these two species to warming trends were most obvious in QDGCs in which they are in sympatry, with Cape rockjumpers showing declines in warming QDGCs where Protea seedeater populations remained relatively stable (Fig. 6).

Cape rockjumpers showed very high costs of evaporative cooling at *T*_air_ between 30 and 38°C, and also experienced elevations in *T*_b_ of similar magnitude to smaller Fynbos species in our respirometry trials. The latter finding is unexpected because larger birds have higher thermal inertia and generally experience lower rates of heat flux, and therefore benefit less from controlled increases in *T*_b_, compared with smaller species (Tieleman and Williams, 1999; Whitfield *et al.*, 2015). This suggests that increases in *T*_b_ above *T*_tb_ may be more difficult to control for Cape rockjumpers, despite expenditure of large amounts of water for evaporative cooling. During summer, *T*_air_ in lowland regions are already frequently well above Cape rockjumpers' *T*_ewl_ (31.2°C) in parts of their range that have experienced rapid warming (e.g. Prince Albert, Great Swartberg Mountains and Western Cape; Fig. 6); conditions that could lead to significant increases in their water demands. The restricted and patchy alpine habitat of the Cape rockjumper, combined with significant warming trends within this habitat, and the Rockjumper's apparent sensitivity to high temperatures, build a strong circumstantial case that recent warming could largely be to blame for population declines in this species. This is further strengthened by the fact that habitat in mountain Fynbos heartland remains relatively untransformed by anthropogenic activity (Turner, 2012), making it unlikely that declines in Cape rockjumpers are linked to habitat destruction. In contrast, climate change appears to be causing increased fire frequencies in mountain Fynbos (Wilson *et al.*, 2010; Kraaij *et al.*, 2013), and Cape rockjumpers show higher reporting rates and densities in recently burnt areas with low vegetation cover (Lee and Barnard, 2015). The large-scale declines observed in Cape rockjumpers therefore further suggest that high temperatures are limiting the ability of this species' to exploit increasing availability of suitable habitat.

In contrast to Cape rockjumpers, evidence of low physiological thermal thresholds in Protea seedeaters is equivocal. Protea seedeaters have low *T*_b_, and low temperature threshold for increases in *T*_b_ (*T*_tb_), but the rate of *T*_b_ increase above this threshold was slow. Costs of evaporative cooling, although higher than in warmer-climate species, are lower than for Cape rockjumpers. However, Protea seedeaters show a lower *T*_ewl_ compared with the closely related Cape canary and Cape siskin. It therefore seems unclear whether current declines in this species result directly from thermal stress linked to climate warming. Their decline may be linked to other habitat changes, perhaps including the changed fire regime, which could affect the supply of *Protea* seeds on which they are reliant (Lee and Barnard, 2014). Conservation action appears to be needed urgently for Cape rockjumpers and Protea seedeaters if their declines are to be understood properly and halted. We need further research to determine the cause of decline of Protea seedeaters and to assess how Cape rockjumpers may best be assisted to cope in the face of continuing climate change. In addition, Cape rockjumpers were an outlier in our study in terms of body mass. Future studies on bird species of similar size to Cape rockjumpers with ranges in the Fynbos biome, but which are not necessarily restricted to cool highlands [e.g. Bokmakierie (*Telophorus zeylonus*), Cape rock thrush (*Monticola rupestris*) and sentinel rock thrush (*Monticola explorator*)] will also allow us better to disentangle body mass effects from cool-climate adaptations in this species.

Not all of the 12 Fynbos species we studied have suffered from reporting rate declines, and some have even shown increases. Two increasing species, Victorin's warbler (a Fynbos endemic) and Cape bunting (a non-endemic resident) show particularly interesting patterns. Like the Cape rockjumper, Victorin's warbler has declined in reporting rate specifically in areas of its range that have warmed significantly (e.g. see Fig. 6). However, warming trends and declines in reporting rate are occurring in less than half of the QDGCs in Victorin's warbler's range, and reporting rates of this species are increasing overall. Unfortunately, our EWL data for Victorin's warbler showed large variation, and we were unable draw inferences about its thermoregulatory costs at high air temperatures. The link between geographical patterns of declines and warming within the range of this species is concerning, and we caution that it might be vulnerable should current population strongholds experience warming in future.

In direct contrast to Victorin's warbler and to all other Fynbos species studied, Cape bunting reporting rates increased in areas of its range that warmed significantly between SABAP1 and SABAP2. However, Cape buntings did not show especially high thermal thresholds for their body size, with *T*_ewl_ not exceptionally high, and a relatively rapid rate of change in *T*_b_ above *T*_tb_. It therefore seems unlikely that Cape bunting is responding to improved thermal conditions within its range, at least in terms of thermal biology of adult birds. Cape buntings do not occupy an especially cold range (MAT > 15°C), so it also seems unlikely, although not impossible, that relaxation of cold stress has caused increases in reporting rates. It is conceivable that warming has changed biotic interactions for Cape buntings, resulting in decreased predation or competition and/or increased food supply. Alternatively, Cape buntings could be responding to entirely different factors not directly linked to warming trends.

One of the pressing questions arising from our study is whether Fynbos birds that occupy cool climatic niches and show low thresholds for heat stress (i.e. Cape rockjumper) have the ability to adjust their physiological responses as they are faced with progressively hotter conditions. Such phenotypic responses can manifest in numerous ways, and individuals that can adjust thermal physiological traits in response to rapid changes in the environment should accrue significant fitness benefits (Piersma and Drent, 2003). Numerous studies have shown that birds can make some physiological adjustments in response to heat or cold acclimation or to season (Dawson, 2003; McKechnie, 2008; Smit and McKechnie, 2010; Smit *et al.*, 2013). For example, many birds display phenotypic flexibility in basal metabolic rate during acclimation to cooler or warmer environments (reviewed by McKechnie and Swanson, 2010). A valuable avenue for future study would be to determine whether Fynbos-endemic birds have the capacity to acclimate to higher values of *T*_air_, and what the physiological costs of these adjustments might be. Our study was conducted early in the Austral summer (October and November 2014) and it could be argued that our study individuals were not sufficiently acclimated to high temperatures. However, despite high *T*_air_ on some days during mid-summer, the minimal and maximal temperatures during mid- to late-summer can also be as low as 1.4 and 13.6°C, respectively, at our study site. Future studies will determine whether heat acclimation is an important response in Fynbos birds inhabiting regions where summer conditions are generally mild; for example, Cape rockjumpers experience *T*_air_ above their *T*_ewl_ less than 10% of the time during mid-summer at Blue Hill Nature Reserve. In addition, captivity stress that birds experience during short-term laboratory studies, such as the present study, might confound important interspecific differences in physiological responses to high temperatures in wild-caught birds. Studies that integrate physiological measurements, e.g. water flux, field metabolic rate and body temperature, with behaviour in free-ranging birds will therefore be an important avenue of future research in temperature-sensitive species (Smit *et al.*, 2013).

### A global context

Evidence that population declines in Fynbos birds under recent climate change are driven by thermal physiology is equivocal on a species level. In a global context, however, we show that cool-climate birds with higher body mass (e.g. Cape sugarbird and Cape rockjumper) do have lower *T*_ewl_ and higher costs of evaporative cooling than similar-sized birds from other biomes. This is almost certainly a result of adaptation of these species to cooler climate envelopes in their ranges. Species such as Cape rockjumper, which occupy cool regions year-round, are likely to have low thermal conductance and/or high insulation properties to reduce thermoregulatory costs (Scholander *et al.*, 1950). Low thermal conductance should lower upper critical thermal limits in endotherms because of reduced rates of heat loss [as has been shown from American pikas (*Ochotona princeps*); MacArthur and Wang, 1973], particularly for medium-to-large species. It has been argued that these physiological attributes could make these mammals highly vulnerable to climate warming, because they are prone to chronic heat stress during summer (Beever *et al.*, 2010), especially in more arid parts of their range (Jeffress *et al.*, 2013). Similar physiological data at high *T*_air_ from birds in cool climates are rare. Tieleman *et al.* (2003) compared thermal physiology between lark species ranging from mesic temperate regions to hot desert regions and showed that two temperate species, skylark (*Alauda arvensis*) and woodlark (*Lullula arborea*), had relatively low *T*_ewl_ (∼35°C) and much greater EWL costs below 40°C compared with the two desert species, Dunn's lark (*Eremalauda dunni*) and hoopoe lark (*Alaemona laudipes*; *T*_ewl_ ∼40°C). Future research should determine whether species restricted to cool regions experience conflicts between cold and warm acclimation demands. For example, does residency in cold climates limit the capacity of endotherms to tolerate and/or acclimate to hot events or hotter regions?

Most physiological parameters scale with body mass (Tieleman and Williams, 2000; McKechnie and Wolf, 2004, 2010), and the lack of a correlation between *T*_ewl_ and body mass in desert birds in our analysis is therefore surprising. This lack of correlation also persists in detailed studies of individual desert bird species (e.g. Whitfield *et al.*, 2015). It seems probable that hot environments have selected for increased thresholds of evaporative cooling in larger desert birds to reduce the thermoregulatory costs associated with high thermal inertia and foraging in hot conditions. The most obvious interpretation of this pattern is that large-bodied temperate birds should be more vulnerable to warming than their desert counterparts, if thermal physiology is the only driver of vulnerability. However, an alternative explanation might be that an adaptive capacity to increase *T*_ewl_ might exist in larger temperate species, but that an adaptive limit in this respect has already been reached by desert birds. Such a possibility would suggest, counter-intuitively, that high *T*_ewl_ in larger desert birds indicates vulnerability to increasing temperatures in future. Differences in thermal physiology between birds from different biomes serve to highlight the complexity of predicting vulnerability to climate change based on physiological data alone.

### Summary

In summary, our study suggests that vulnerability to climate change may not always be determined primarily by physiological thermal thresholds, despite these often being cited as the most fundamental driver (Kearney and Porter, 2009; Monahan, 2009; Kearney *et al.*, 2010; Angert *et al.*, 2011; Sunday *et al.*, 2012; Khaliq *et al.*, 2014). For example, of all the Fynbos bird species we studied, only one (Cape rockjumper) clearly appears to be vulnerable to warming as a result of low physiological thermal thresholds. Other factors, such as trade-offs between physiological and behavioural costs of thermoregulation (Sinervo *et al.*, 2010; du Plessis *et al.*, 2012; Cunningham *et al.*, 2013, 2015; Smit and McKechnie, 2015), altered biotic interactions or changes in variables other than temperature under climate change (e.g. precipitation or fire regimes; Lee and Barnard, 2014), may also be important. While thermal physiology almost certainly sets absolute upper limits on the ability of organisms to cope with rising temperatures (at least within the bounds of phenotypic plasticity and adaptation), these other factors may limit populations before physiological thresholds are reached. Despite this, the striking similarity between patterns of decline in association with temperature in our study and the studies by Jiguet *et al.* (2006, 2010) of north temperate birds suggests that many of these alternative drivers will co-vary with climate change. Therefore, the observation holds that, at least in temperate zones, cool-climate birds appear most vulnerable to warming; although the mechanisms driving decline remain in many cases unknown.

## Supplementary material

Supplementary material is available at *Conservation Physiology* online.

## Funding

This research was funded by the DST/NRF Centre of Excellence at the Percy FitzPatrick Institute of African Ornithology, University of Cape Town, and a Nelson Mandela Metropolitan University research grant to B.S. The work on animals in this study was approved by the Science Faculty Animal Ethics Committee, University of Cape Town (#2013/V23/PR).

## Supplementary Material

Supplementary Data
