# Supplementary material for: The role of thermal physiology in recent declines of birds in a biodiversity hotspot
Source: Conserv Physiol. 2015 Nov 13;3(1):cov048. doi: 10.1093/conphys/cov048 (PMC4778484; doi:10.1093/conphys/cov048)
Supplement: Supplementary Data [file cov048supp.zip › cov048supp.docx]

**Supporting information**

**Materials and methods**

***Gas exchange measurements***

Birds caught at Blue Hill Nature Reserve were placed individually in respirometry chambers constructed from airtight plastic containers (1.9, 4 or 6 L, depending on the size of the bird). A 0.5 cm layer of mineral oil was placed in the bottom of each chamber to prevent evaporation from faeces, thus excluding these as sources of water in measurements. Birds were placed on a wire mesh platform secured 5 to 10 cm above the oil layer, with large enough holes to allow faeces to fall through. The respirometry chamber containing a bird was placed within an insulated environmental chamber constructed by lining the interior of a 100 L cooler box with copper tubing (Smit and McKechnie, 2010a). The temperature within the respirometry chamber was regulated by pumping temperature-controlled water from a circulating water bath (FRB22D, Lasec, Cape Town, South Africa) through the copper tubing in the environmental chamber using a small water pump. An electric fan was used to ensure air circulation within the cooler box. Air temperature within the respirometry chamber was measured using a thermistor probe (model TC100, Sable Systems, Las Vegas, NV, USA) inserted into the chamber through a small hole in the lid.

All respirometry experiments were conducted during the day (i.e. sunrise to sunset), which is the active phase of all 12 species used in our study. Prior to each experiment, birds were weighed to the nearest 0.01 g. Nectarivores were provided with sugar water (25% sucrose solution) directly before the experiment, given the high gut passage rates often observed in these birds (Mbatha *et al.*, 2002), to prevent dehydration and reduce fasting periods. We trimmed tail feathers of birds with particularly long tails so that the tail extending through the wire mesh did not reach the oil layer. These included certain individuals from Cape Sugarbird, Orange-breasted Sunbird, Malachite Sunbird, Victorin’s Warbler, Cape Grassbird and Familiar Chat.

Each bird was placed individually into a suitably-sized respirometry chamber. The lid of the environmental chamber was closed immediately, creating a darkened environment to minimise distress in the bird. Birds were monitored constantly for signs of distress during the trials using a closed-circuit surveillance camera and an infra-red light source inside the environmental chamber. Soon after the chamber was darkened the birds generally adopted a relaxed pose as observed through night vision video-feed. During respirometry experiments, birds experienced a ramped profile of five controlled temperature treatments (starting at a low temperature), each lasting approximately 20-30 minutes. Such a ramped profile is appropriate since this is how birds generally experience temperature changes in the wild. Air temperature was only increased if the bird remained calm during the test period (assessed using the live video feed together with metabolic gas traces, see below). Incremental changes in Tair occurred gradually and typically took about 15 to 20 min for Tair within the chamber to stabilise at the new level (2° – 4 °C higher).

Atmospheric air from outside the building was pushed through the system using an air compressor (MOA-P101-CD, GAST Manufacturing, Inc., Michigan, USA). Water vapour was scrubbed from the air using a silica gel and a drierite column connected in series. The flow rate into the respirometry chamber was regulated using an FMA-series mass flow controller (Omega, Bridgeport, NJ, USA) calibrated using a flow-bubble meter. Flow rates of between 1.0 and 5.5 L min-1 were used throughout measurements, depending on the species and Tair. These flow rates were selected to ensure low water vapour pressures in the chamber, thus ensuring high vapour pressure deficits (>5 kPa) to maximise the efficiency of evaporative cooling. Maintaining low humidity in the chamber appeared to significantly reduce the likelihood of stress in the birds at higher air temperatures. Excurrent air from the respirometry chamber and a reference air supply (baseline air sampled downstream of scrubbers) was sub-sampled using an SS1 sub-sampler (Sable Systems), and a TR-RM8 Respirometry Multiplexer (V3, Sable Systems). Baseline water vapour pressure (kPa) and CO2 and O2 concentrations (%) were obtained from the reference air channel for at least 10 min before placing the bird into the respirometry chamber, as well as for 5 min following every experimental temperature period during the trial by manually switching between airstreams. Sub-sampled air first passed through a water analyser (RH-300, Sable Systems) to measure water vapour pressure. Air was then pulled through a silica gel column to remove water vapour before passing through a CO2 analyser (Ca-10a, Sable Systems) to measure the CO2 concentration. At the beginning of the study both the CO2 and water analysers were zeroed using pure nitrogen. The CO2 analyser was calibrated against an analytically certified gas with a known CO2 concentration of 1 500 ppm. The water analyser was calibrated by calculating the water vapour pressure of air saturated at 16°C. The air sample then passed through a soda lime/silica gel column to remove CO2 and water vapour, respectively, before entering an O2 analyser (Fc-10a, Sable Systems) to measure the fractional O2 concentration. The O2 analyser was spanned to a fractional O2 concentration of 20.95% at the start of each experimental trial. Outputs from these three gas analysers were digitised using a Universal Interface II system (Sable Systems) and recorded with a sampling interval of 1 second using Expedata data acquisition software (ExpeData Data Acquisition & Analysis version 1.1.18, Sable Systems) loaded onto a personal computer.

***Body temperature measurements***

Body temperature (Tb, °C) of birds was measured throughout metabolic trials using a lubricated fine-gauge Teflon-coated Cu-Cn thermocouple (IT-118, Physitemp, Clifton, NJ). The thermocouple was inserted into the cloaca of the bird to a depth at which a slight withdrawal did not result in a change in the temperature reading (between 0.5 and 2 cm, depending on the size of the bird; Smit and McKechnie, 2010b). The thermocouple was secured by attaching the wire to the feathers immediately behind the cloaca, using adhesive tape and a small wire paperclip. Generally, the thermocouple caused no detectable discomfort in the birds and stayed intact throughout the experimental trial. However, some individuals removed the thermocouple with their beaks during respirometry runs. The sample size of Tb measurements is thus smaller than for EWL measurements. Outputs from the thermocouple were digitised using a TC-1000 thermocouple meter (Sable Systems) linked to Expedata software on personal computer.

***Experimental protocol***

Birds often allow controlled elevations in Tb above normal levels when exposed to heat (Smit *et al.*, 2013; Tieleman and Williams, 1999), however lethal Tb (generally from 45° to 47 °C in birds) had to be avoided (Dawson, 1954). Therefore, Tb as well as , and EWL were monitored continually throughout experimental trials using the Expedata data acquisition software. Concurrently, activity (calm, moving/looking around or jumping) and heat dissipation (panting or wing spreading) behaviours were noted and recorded every 5 min. If birds showed any signs of restlessness that resulted in a sudden increase in Tb (above 43 °C), the trial ceased and the bird was removed from the chamber. Reported values are thus associated with periods of approximately stable RMR and EWL.

***Release and/or housing of birds***

After the completion of the experimental trial, the temperature was reduced to allow the chamber to cool down to approximately room temperature (25 °C). The bird was then removed from the chamber and the thermocouple carefully extracted. The bird was weighed, given water to drink and then placed into a cloth bag. Nectarivores were additionally fed a 25% sucrose solution on removal from the chamber. Birds were, as far as possible, released directly after trials at the site where they were captured. Birds for which runs finished close to sunset were kept overnight in small shade cloth cages (30x40x50 cm). Cages were placed in a separate, quiet room with room temperature maintained at approximately 25 °C and were protected from noise and other disturbances. Under these circumstances, birds had access to *ad libitum* water and food appropriate to the species (25% sucrose solution for nectarivores, birdseed for granivores, mealworms for insectivores) to ensure they maintained energy and water balance, before being released the following morning at their capture site. Additionally, in the event that more than one bird was captured at the same time, while the first bird was in the respirometry chamber, the others were housed under conditions described above. Birds were held under these circumstances for no more than 48 hours.

***Data analysis***

Corrections for lag in the O2, CO2 and water vapour measurements, as well as corrections for drift in the O2 measurements, were made using the appropriate regression analyses in Expedata. Due to the large variability of O2 readings at high flow rates, the respiratory exchange ratio (RER) of each bird had to be assumed based on its dietary guild and an estimation of the time since its last meal. Nectarivores were assumed to metabolise carbohydrates (RER = 1.00), whereas all other species were assumed to metabolise lipids [RER = 0.71, (Walsberg and Wolf, 1995)]. was then calculated from the equation RER = /(Walsberg and Wolf, 1995).Mass-specific resting metabolic rate (RMR, reported as rate of metabolic heat production, J g-1 h-1) was calculated from steady state traces of and the calculated values, using equation 9.8 in (Lighton, 2008). Mass-specific evaporative water loss (EWL, reported as rate of water vapour production, mgg-1h-1) was calculated from steady state EWL traces in Expedata, using equation 9.9 in (Lighton, 2008). In all cases, the lowest 5-10 min mean at each experimental temperature was considered to be indicative of resting values. All and values were corrected to STPD (standard temperature, pressure, dry). About mid-way through the study the infra-red detector of the CA-10 analyser failed, resulting in smaller differences between sample and baseline values during some of the runs. Resting metabolic rate values calculated from these runs were distinctly lower (30 – 60%) than expected RMR for the species and were excluded from analyses.

**Phylogenetic analyses and interspecific comparisons**

Phylogenetic independence tests take into account that traits may be similar due to relatedness (Garland and Adolph, 1994). Phylogenetic least squares (PGLS) analyses using *ape* (Paradis *et al.*, 2004) and *caper* (Orme, 2013) packages in R were performed on the global multispecies dataset (34 species) to verify the outcomes of all conventional glms. We sampled 100 hypothetical phylogenies from http://www.birdtree.org (Jetz *et al.*, 2012) using the Hackett *et al.* (2008) phylogeny as backbone (Fig. S1). Mesquite (Maddison and Maddison, 2011) was used to generate the maximum consensus tree for phylogenetic independent analyses. We ran models with both lambda and kappa branch length transformations. AICc values for models using each transformation were generally similar (∆AICc< 2) and models with lambda branch length transformations are presented. Null models of Tewl, log10slope of EWL and log10 Mb from the global analysis were used to establish phylogenetic signal (a measure of how strongly correlations in traits reflect shared ancestry), however phylogenetic signals were not detected for any of the above variables; slope of EWL above Tewl (K = 0.671, p = 0.314), Mb (K = 0.0, p = 1.0), and Tewl (K = 0.557, p = 0.078).

Blomberg *et al.* (2003) suggest a minimum of 20 species in data sets to detect phylogenetic signal and to perform accurate branch length transformations using PGLS. We only had 12 species for analyses including Blue Hill Nature Reserve species only. We therefore only present conventional analyses for these species. All the species studies at Blue Hill Nature Reserve belong to the order passeriformes, although Cape Rockjumper (family Chaetopidae, two species; Cape and Drakensberg Rockjumper) represents a phylogenetically ancient passerine lineage closely related to the Rockfowl (family Picatharthidae).

**Results**

***Sample sizes***

Each individual could only be exposed to a maximum of five experimental temperatures (half of the full temperature regime) during a single respirometry trial, because birds had to remain at each temperature for roughly 30 min, it took 15 to 20 min for each new temperature to stabilise, and the total length of time spent inside the chamber during a single measurement session could not exceed four hours for ethical reasons. We aimed to run ten individuals per species in order to obtain sufficient data across the full range of temperatures for each species during our field season. During the ten week field period, 393 individuals of the 12 target species were captured. 100 of these were successfully processed in the respirometry chamber and data were collected for these individuals.

***Thermal physiological attributes of Blue Hill Nature Reserve species***

**Evaporative water loss**

In all 12 species, the rate of mass-specific evaporative water loss (EWL) remained relatively constant at low air temperatures (Tair) up to a certain Tair threshold (Tewl), after which it increased linearly with increasing Tair (Fig. S3). Tewl inflection points were statistically significant (i.e. the slopes above and below Tewl were significantly different) for all species, except Victorin’s Warbler (which still shows an increase, but not significant due to large variation, Table S1). Southern Double Collared Sunbird had the highest Tewl inflection point (37.5° ± 1.0 °C), which was 6.2 °C higher than that of Cape Rockjumper with the lowest inflection point (31.3° ± 0.6 °C). The rate of EWL increase (i.e. the slope of EWL) above Tewl was significantly greater than zero for 10 species. The three small sunbird species had the steepest slopes above their respective Tewl (> 4mg g-1 h-1°C-1). Cape Rockjumper showed the lowest rate of EWL increase above its Tewl (1.20 ± 0.10mg g-1 h-1 °C-1).

**Panting**

The mean temperature at which individual birds started to show panting behaviour (Tpant) was 35.1° ± 0.2 °C. Orange-breasted Sunbird had the highest mean Tpant (37.8° ± 0.6 °C), whereas Cape Rockjumper had the lowest (33.6° ± 0.7 °C) (Table S2).

**Resting metabolic rate**

Three species (Victorin’s Warbler, Cape Bunting and Cape Grassbird) showed a significant negative trend in mass-specific resting metabolic rate (RMR) across the entire range of air temperatures (Tair) to which they were exposed (Fig.S4). Five species showed a significant lower inflection point in the relationship between RMR and Tair (Fig. S4). In some of these species RMR still showed a significant decreasing trend above these inflection points. For example, in Cape Sugarbird, RMR decreased at a significant rate of -0.81 ± 0.25 J g-1 h-1°C-1 (t = -3.16, p = 0.004) at temperatures above the inflection point of 25.7° ± 1.1 °C (below this point RMR increased steeply, although this was based on a small number of data points). In Familiar Chat, RMR increased significantly above Tair 31.5 °C at 2.35 J g-1 h-1 °C-1 (Table S3). Other species showed no significant change in slope above or below their respective inflection points, e.g. Cape Siskin, Southern Double-collared Sunbird and Cape Canary. Cape Siskin, Protea Seedeater, Cape Rockjumper, Cape Canary, and Cape Bunting showed no significant relationship between RMR and Tair over the Tair tested (Table S3). We therefore could not identify reliable upper limits to thermoneutrality, using RMR, for the study species. Values of RMR at *T*air = 30°C and *T*air = 38°C are displayed in Table S4.

**Body temperature**

All species, except Cape Canary, showed an increase in body temperature (Tb) with air temperature (Tair). Eight species showed a significant inflection point in the relationship between Tb and Tair, after which Tb increased significantly at increasing Tair (Fig.S5). Southern Double-collared Sunbird had the highest inflection point (35.2° ± 0.6 °C) and the steepest slope above this point (0.68 ± 0.09 °C Tb°C-1 Tair, t = 7.50, p = 0.006). Cape Sugarbird had the lowest inflection point (30.7° ± 1.0 °C), and increased Tb at a rate of 0.36 °C °C-1 Tair. Cape Canary showed the most stable Tb with lowest rate of Tb increase above its inflection point (0.02 ± 0.04, t = 5.66, p = 0.001). Mean Tb values ranged from 38.2° to 40.3 °C at Tair = 30 °C (Table S5).

**Figures**

**Figure S1:** Phylogeny of the 12 bird species in which various physiological parameters were investigated at Blue Hill Nature Reserve, Western Cape, South Africa. This phylogenetic tree represents the majority consensus treefrom100 hypothetical phylogenies that were sampled for each data set from http://www.birdtree.org (Jetz *et al.*, 2012) using the Hackett *et al.* (2008) phylogeny as backbone.


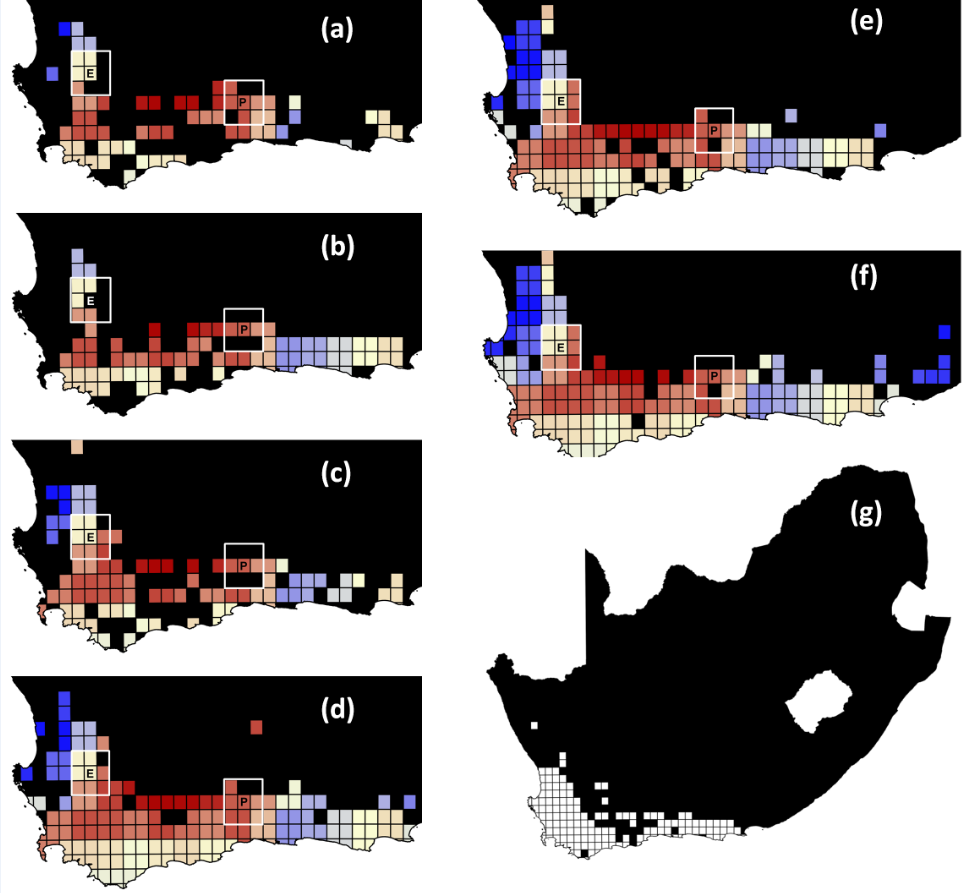


**Figure S2:** Maps of the Fynbos biome. (a) – (f) show the distribution of the six Fynbos endemic passerines, and rates of warming within each QDGC of their range between 1987 and 2010. Red cells indicate warming trends, blue cells indicate cooling trends, pale yellow cells indicate temperatures remained stable across the time period. Intensity of colour indicates strength of the warming trend (deeper colours denote stronger warming/cooling). Coloured cells indicate the species was present in either SABAP1 or SABAP2, or in both periods. Black cells indicate that the species was not present in either survey period. (a) Cape Rockjumper, (b) Victorin’s Warbler, (c) Protea Seedeater, (d) Orange-breasted Sunbird, (e) Cape Siskin, (f) Cape Sugarbird. (g) Map of the location of the Fynbos biome within South Africa: white QDGCs indicate land-cover comprises 50% or more Fynbos vegetation, by area. Data extracted from SABAP2 (www.sabap2.adu.org.za), the University of Delaware Gridded Climate Database (version 3.01; Matsuura and Willmott, 2012), and South African National Landcover shape files (version 1.1; van den Berg *et al.*, 2008). The white blocks represent nine QDGCs surrounding the Excelsior (E) and Prins Albert (P) weather stations described in Figure 6 (main text).

**Figure S3:** Mass-specific evaporative water loss (EWL, mg g-1h-1) in 12 species studied at Blue Hill Nature Reserve over a range of air temperatures (Tair, °C). Segmented regressions were used to estimate the inflection point (Tewl, °C) of the relationship between EWL and Tair. Data below and above Tewl are indicated by open and filled circles, respectively. Linear mixed effects regression analyses were performed on data above inflection points. Trendlines represent significant relationships (p < 0.05). See Table S1 for statistics.

**Figure S4:** Mass-specific resting metabolic rate (RMR) in 11 species studied at Blue Hill Nature Reserve over a range of air temperatures (Tair, °C). Segmented regressions were used to estimate the inflection point (°C) of the relationship between RMR and Tair. Linear mixed effects regression analyses were performed on data above inflection points. Clear symbols indicate values which are below the upper critical limit of TNZ. See Table S3 for statistics.

**Figure S5:** Body temperature (Tb, °C) in 12 species studied at Blue Hill Nature Reserve over a range of air temperatures (Tair, °C). Segmented regressions were used to estimate the inflection point (°C) of the relationship between Tb and Tair. Linear mixed effects regression analyses were performed on data above inflection points. In cases where inflection points were not significant, and there appeared to be no inflection in the data, linear mixed effects regression analyses were performed on the entire dataset for that species. Solid lines represent significant relationships (p<0.05). In the case of significant inflection points, clear symbols indicate values below the inflection point. See Table S5 for statistics.

**Tables**

**Table S1:** Evaporative water loss (EWL) measures for all 12 species studied at Blue Hill Nature Reserve, South Africa, showing the sample size (n) of each species, the outcomes of the Davies’ Test for a change in slope, the inflection point in the relationship between air temperature (Tair) and EWL (Tewl, °C) as determined by the segmented model, as well as the outcomes of the linear mixed effects model for the slope (Beta, mg g-1h-1°C-1) of the segment above the Tewl, regardless of whether this inflection point was significant for the particular species.

|  |  | **Davies’ Test for change in slope** | | **Tewl (°C, segmented model)** | | **Slope (mg g-1 h-1 °C-1, linear mixed effects model)** | | | | | | |
| --- | --- | --- | --- | --- | --- | --- | --- | --- | --- | --- | --- | --- |
| **Species** | **n** | **P** | **Significance** | **Estimate** | **s.e.** | **Intercept** | **Beta** | **s.e.** | **Df** | **t** | **P** | **Significance** |
| Cape Sugarbird | 11 | 0.000 | *** | 34.6 | 0.6 | -47.39 | 1.56 | 0.18 | 10 | 8.73 | 0 | *** |
| Orange-breasted Sunbird | 10 | 0.006 | ** | 36.7 | 1.0 | -152.46 | 4.51 | 0.65 | 4 | 6.89 | 0.002 | ** |
| Cape Siskin | 10 | 0.000 | *** | 35.6 | 0.5 | -118.08 | 3.56 | 0.25 | 7 | 14.48 | 0 | ** |
| Protea Seedeater | 9 | 0.000 | *** | 33.5 | 0.6 | -68.92 | 2.28 | 0.22 | 12 | 10.26 | 0 | *** |
| Victorin's Warbler | 6 | 0.091 |  | 34.7 | 1.3 | -44.81 | 1.68 | 0.35 | 1 | 4.86 | 0.129 | NS |
| Cape Rockjumper | 10 | 0.000 | *** | 31.3 | 0.7 | -34.49 | 1.20 | 0.10 | 20 | 11.96 | 0 | *** |
| Malachite Sunbird | 5 | 0.000 | *** | 35.5 | 0.6 | -128.97 | 4.03 | 0.23 | 4 | 17.56 | 0.000 | *** |
| Southern Double-collared Sunbird | 10 | 0.000 | *** | 37.5 | 0.2 | -515.68 | 14.24 | 6.06 | 2 | 2.35 | 0.143 | NS |
| Cape Canary | 4 | 0.000 | *** | 34.0 | 0.6 | NA | NA | NA | NA | NA | NA |  |
| Cape Bunting | 10 | 0.000 | *** | 35.0 | 0.4 | -87.98 | 2.74 | 0.30 | 7 | 9.21 | 0.000 | *** |
| Cape Grassbird | 5 | 0.000 | *** | 33.5 | 0.5 | -54.14 | 1.78 | 0.16 | 7 | 11.36 | 0.000 | *** |
| Familiar Chat | 10 | 0.000 | *** | 33.5 | 0.5 | -68.10 | 2.18 | 0.28 | 9 | 7.89 | 0.000 | *** |

* p< 0.05, ** p< 0.01,*** p< 0.001

**Table S2:** Mean temperature at which birds started panting (Tpant ± s.e., °C) for 12 species at Blue Hill Nature Reserve, South Africa.

| **Species** | **n** | **n pant** | **Mean Tpant ± s.e. (°C)** |
| --- | --- | --- | --- |
| Cape Sugarbird | 11 | 11 | 35.2 ± 0.3 |
| Orange-breasted Sunbird | 10 | 6 | 37.8 ± 0.6 |
| Cape Siskin | 10 | 8 | 35.8 ± 0.5 |
| Protea Seedeater | 9 | 9 | 33.8 ± 0.1 |
| Victorin's Warbler | 6 | 6 | 33.9 ± 0.4 |
| Cape Rockjumper | 10 | 8 | 33.6 ± 0.7 |
| Malachite Sunbird | 5 | 4 | 35.8 ± 0.2 |
| Southern Double-collared Sunbird | 11 | 5 | 37.2 ± 0.5 |
| Cape Canary | 4 | 1 | 34.1 (NA) |
| Cape Bunting | 10 | 8 | 34.5 ± 0.7 |
| Cape Grassbird | 5 | 5 | 34.1 ± 0.4 |
| Familiar Chat | 10 | 6 | 36.5 ± 0.2 |

**Table S3:**Resting metabolic rate (RMR) measures for all 12 species studied at Blue Hill Nature Reserve, South Africa, showing the sample size (n) of each species, the outcomes of the Davies’ Test for a change in slope, the inflection point (°C) in the relationship between air temperature (Tair) and RMR as determined by the segmented model. Inflection points that are representative of upper (U) or lower (L) inflection points of the thermoneutral zone (TNZ) are indicated as superscripts under the estimate. In cases where an upper inflection point was identified, the outcomes of the linear mixed effects model for the slope (Slope, J g-1 h-1 °C-1) of the upper segment are listed (if applicable).

|  |  | **Davies’ Test for change in slope** | | **RMR inflection point (°C, segmented model)** | | **Slope (J g-1 h-1 °C-1, linear mixed effects model)** | | | | | | |
| --- | --- | --- | --- | --- | --- | --- | --- | --- | --- | --- | --- | --- |
| **Species** | **n** | **p** | **Significance** | **Estimate** | **s.e.** | **Intercept** | **Beta** | **s.e.** | **Df** | **t** | **p** | **Significance** |
| Cape Sugarbird | 6 | 0.047 | * | 25.7L | 1.1 |  |  |  |  |  |  |  |
| Orange-breasted Sunbird | 6 | 0.001 | ** | 37.0U | 0.8 | NA | NA |  |  |  |  |  |
| Cape Siskin | 10 | 0.047 | * | 27.3L | 1.1 |  |  |  |  |  |  |  |
| Protea Seedeater | 5 | 0.405 |  | 38.0 | 0.1 |  |  |  |  |  |  |  |
| Victorin's Warbler | 4 | 1 |  | 34.2 | 4.4 |  |  |  |  |  |  |  |
| Cape Rockjumper | 7 | 0.406 |  | 22.3 | 0.1 |  |  |  |  |  |  |  |
| Malachite Sunbird | 0 |  |  |  |  | NA | NA |  |  |  |  |  |
| Southern Double-collared Sunbird | 6 | 0.049 | * | 37.6U | 0.5 | NA | NA |  |  |  |  |  |
| Cape Canary | 3 |  |  |  |  | NA | NA |  |  |  |  |  |
| Cape Bunting | 3 | 0.263 |  | 37.3 | 1.4 |  |  |  |  |  |  |  |
| Cape Grassbird | 4 | 0.133 |  | 27.2 | 0.6 |  |  |  |  |  |  |  |
| Familiar Chat | 6 | 0.000 | *** | 31.5U | 0.8 | -20.95 | 2.35 | 0.67 | 7 | 3.53 | 0.010 | ** |

* p< 0.05, ** p< 0.01, *** p< 0.001, NA: too few data points

**Table S4:** Mass-specific evaporative water loss (EWL, mg g-1 h-1), resting metabolic rate (RMR, J g-1 h-1) and body temperature (Tb, °C) at air temperature of 30 °C for each of the 12 species studied at Blue Hill Nature Reserve, South Africa, showing mean ± 1 standard error for each measure, the number of individuals included in the calculation of these values (n).

| **Species** | **EWL (mg g-1 h-1)** | **RMR (J g-1 h-1)** | **Tb (°C)** |
| --- | --- | --- | --- |
| Cape Sugarbird | 5.4 ± 0.5 (6) | 49.7 ± 6.3 (3) | 39.4 ± 0.3 (5) |
| Orange-breasted Sunbird | 9.8 ± 0.9 (5) | 73.5 ± 3.7 (4) | 40.3 ± 0.4 (3) |
| Cape Siskin | 7.0 ± 1.7 (3) | 72.3 (2) | 40.2 ± 0.4 (3) |
| Protea Seedeater | 6.0 ± 0.8 (5) | 76.3 ± 10.9 (3) | 39.3 ± 0.2 95) |
| Victorin’s Warbler | 8.4 ± 1.1 (3) | 55.2 (1) | 39.7 ± 0.0 (2) |
| Cape Rockjumper | 2.6 ± 0.2 (5) | 36.7 ± 1.5 (4) | 39.3 ± 0.2 (3) |
| Malachite Sunbird | 8.9 ± 1.2 (2) | NA (0) | 38.2 (1) |
| Southern Double-collared Sunbird | 10.7 ± 1.4 (5) | 67.3 (1) | 39.4 ± 0.1 (3) |
| Cape Canary | 9.2 ± 1.8 (3) | 55.2 ± 2.5 (3) | 39.9 ± 0.2 (3) |
| Cape Bunting | 6.0 ± 0.5 (7) | 57.8 ± 4.7 (2) | 39.4 ± 0.2 (7) |
| Cape Grassbird | 5.5 ± 0.5 (5) | 72.1 ± 7.0 (4) | 39.6 ± 0.2 (2) |
| Familiar Chat | 5.1 ± 0.4 (6) | 59.2 ± 3.4 (3) | 40.0 ± 0.2 (6) |

NA: too few data points

**Table S5:** Body temperature (Tb) measures for all 12 species studied at Blue Hill Nature Reserve, South Africa, showing the sample size (n) of each species, the outcomes of the Davies’ Test for a change in slope, the inflection point (°C) in the relationship between air temperature (Tair) and Tb as determined by the segmented model, as well as the outcomes of the linear mixed effects model for the slope (Beta, mgg-1 h-1 °C-1) of the segment above significant inflection points. In cases where the Davies’ Test showed no significant change in slope and therefore no significant Tb inflection point, the slope was calculated for the entire dataset and not only for the segment above the point, and these values are shown here.

|  |  | | **Davies’ Test for change in slope** | | **Tb inflection point (°C, segmented model)** | | **Slope (°C Tb°C-1Tair, linear mixed effects model)** | | | | | | |
| --- | --- | --- | --- | --- | --- | --- | --- | --- | --- | --- | --- | --- | --- |
| **Species** | **n** | **P** | | **Significance** | **Estimate** | **s.e.** | **Intercept** | **Beta** | **s.e.** | **Df** | **t** | **p** | **Significance** |
| Cape Sugarbird | 11 | 0.000 | | *** | 30.7 | 1.0 | 27.77 | 0.36 | 0.03 | 21 | 13.00 | 0 | **** |
| Orange-breasted Sunbird | 8 | 0.000 | | *** | 34.7 | 0.4 | 21.97 | 0.52 | 0.03 | 9 | 15.45 | 0 | **** |
| Cape Siskin | 9 | 0.066 | |  | 34.3 | 1.3 | 35.39 | 0.17 | 0.02 | 28 | 7.07 | 0 | **** |
| Protea Seedeater | 7 | 0.000 | | *** | 30.9 | 0.9 | 30.88 | 0.27 | 0.05 | 14 | 5.80 | 0 | **** |
| Victorin's Warbler | 5 | 0.187 | |  |  |  | 35.35 | 0.16 | 0.04 | 14 | 4.30 | 0.001 | *** |
| Cape Rockjumper | 4 | 0.000 | | *** | 32.1 | 0.8 | 26.90 | 0.38 | 0.03 | 5 | 14.97 | 0 | **** |
| Malachite Sunbird | 1 | NA | | NA | NA | NA | NA | NA | NA | NA | NA | NA | NA |
| Southern Double-collared Sunbird | 8 | 0.000 | | *** | 35.2 | 0.6 | 15.44 | 0.68 | 0.09 | 5 | 7.50 | 0.001 | *** |
| Cape Canary | 3 | 0.929 | |  |  |  | NA | NA | NA | NA | NA | NA |  |
| Cape Bunting | 10 | 0.000 | | *** | 32.6 | 0.8 | 25.72 | 0.42 | 0.02 | 13 | 20.03 | 0 | **** |
| Cape Grassbird | 2 | 0.047 | | * | 30.8 | 0.9 | 31.91 | 0.25 | 0.04 | 4 | 5.66 | 0.005 | ** |
| Familiar Chat | 8 | 0.000 | | *** | 33.3 | 0.7 | 26.41 | 0.41 | 0.05 | 5 | 8.26 | 0.000 | *** |

* p< 0.05, ** p< 0.01, *** p< 0.001, NA: too few data points

**Table S6:** A summary of the physiological data from 34 species used in the global multispecies comparative analysis. This includes data from the 12 species studied at Blue Hill Nature Reserve for this study, data for two doves species studied by Whitfield *et al.*(unpublished data), and three passerines by Whitfield *et al.* (2015) and data extracted from 17 species in the analyses of (McKechnie and Wolf, 2010). The table includes common and scientific names of species, the broad climatic zone in which the majority of the species’ distribution is centred (classified according to Köppen 1990, updated version: (Kottek *et al.*, 2006), Blue Hill Nature Reservemean body mass (Mb, g), the mean inflection point in the relationship between evaporative water loss (EWL) and air temperature (Tewl, °C), the mean slope of the line above this inflection point (EWL slope, mg g-1 h-1 °C-1).

| **Species** |  | **Climatic zone** | **Mb (g)** | **log10 Mb** | **Tewl(°C)** | **EWL slope (mg g-1 h-1 °C-1)** | **Source** |
| --- | --- | --- | --- | --- | --- | --- | --- |
| Cape Bunting | *Emberiza capensis* | Temperate | 19.8 | 1.3 | 35 | 2.74 | Present study |
| Cape Canary | *Serinus canicollis* | Temperate | 14.9 | 1.2 | 34 | 3.63 | Present study |
| Cape Grassbird | *Sphenoeacus afer* | Temperate | 30.0 | 1.5 | 33.5 | 1.78 | Present study |
| Cape Rockjumper | *Chaetops frenatus* | Temperate | 53.7 | 1.7 | 31.3 | 1.2 | Present study |
| Cape Siskin | *Serinus totta* | Temperate | 12.6 | 1.1 | 35.6 | 3.56 | Present study |
| Cape Sugarbird | *Promerops cafer* | Temperate | 36.2 | 1.6 | 34.6 | 1.56 | Present study |
| Malachite Sunbird | *Nectarinia famosa* | Temperate | 15.2 | 1.2 | 35.5 | 4.03 | Present study |
| Orange-breasted Sunbird | *Anthobaphes violacea* | Temperate | 9.4 | 1.0 | 36.7 | 4.51 | Present study |
| Protea Seedeater | *Crithagra leucoptera* | Temperate | 19.8 | 1.3 | 33.5 | 2.28 | Present study |
| Southern Double-collared Sunbird | *Nectarinia chalybea* | Temperate | 7.5 | 0.9 | 37.5 | 14.24 | Present study |
| Victorin's Warbler | *Bradypterus victorini* | Temperate | 17.1 | 1.2 | 34.7 | 1.68 | Present study |
| Familiar Chat | *Cercomela familiaris* | Temperate | 20.3 | 1.3 | 33.5 | 2.18 | Present study |
| Bridled Titmouse | *Baeolophus wollweberi* | Temperate | 10.5 | 1.0 | 37.9 | 5.07 | Weathers and Greene, 1998 |
| Juniper Titmouse | *Baeolophus ridgwayi* | Temperate | 17.0 | 1.2 | 36.3 | 2.91 | Weathers and Greene, 1998 |
| Black-rumped Waxbill | *Estrilda troglodytes* | Tropical | 6.0 | 0.8 | 38.7 | 6.28 | Cade *et al.*, 1965 |
| Dusky Munia | *Lonchura fuscans* | Tropical | 9.5 | 1.0 | 34.7 | 2.79 | Weathers, 1977 |
| Gouldian Finch | *Erythrura gouldiae* | Tropical | 17.1 | 1.2 | 36.4 | 3.18 | Burton and Weathers, 2003 |
| Monk Parakeet | *Myiopsitta monachus* | Tropical | 80.4 | 1.9 | 34.3 | 1.83 | Weathers *et al.*, 1975 |
| Variable Seedeater | *Sporophila corvina* | Tropical | 10.9 | 1.0 | 37.9 | 4.48 | Weathers, 1997 |
| Laughing Dove | *Stigmatopelia senegalensis* | Desert | 86.5 | 1.9 | 39.9 | 1.87 | Whitfield *et al.* unpublished data |
| Namaqua Dove | *Oena capensis* | Desert | 35.8 | 1.6 | 40.8 | 2.50 | Whitfield *et al.* unpublished data |
| Scaly-feathered Weaver | *Sporopipes squamifrons* | Desert | 10.6 | 1.0 | 39.3 | 3.67 | Whitfield *et al.*, 2015 |
| Sociable Weaver | *Philetairus socius* | Desert | 24.9 | 1.4 | 42.8 | 3.17 | Whitfield *et al.*, 2015 |
| White-browed Sparrow-weaver | *Plocepasser mahali* | Desert | 41.1 | 1.6 | 40.2 | 4.01 | Whitfield *et al.*, 2015 |
| Budgerigar | *Melopsittacus undulates* | Desert | 33.7 | 1.5 | 37.9 | 3.46 | Weathers and Schoenbaechler, 1976 |
| Diamond Dove | *Geopelia cuneata* | Desert | 38.0 | 1.6 | 39.4 | 2.44 | Schleucher, 1999 |
| Dune Lark | *Certhilauda erythrochlamys* | Desert | 27.3 | 1.4 | 36.5 | 5.34 | Williams, 1999 |
| Dunn's Lark | *Eremalauda dunni* | Desert | 20.6 | 1.3 | 39.8 | 4.54 | Tieleman and Williams, 2002 |
| Greater Hoopoe-lark | *Alaemona laudipes* | Desert | 37.7 | 1.6 | 38.6 | 4.32 | Tieleman and Williams, 2002 |
| Spinifex Pigeon | *Geophaps plumifera* | Desert | 89.0 | 1.9 | 37.7 | 1.49 | Withers and Williams, 1990 |
| Spinifexbird | *Eremiornis carteri* | Desert | 12.0 | 1.1 | 37.3 | 4.10 | Ambrose *et al.*, 1996 |
| Spotted Nightjar | *Eurostopodus argus* | Desert | 88.0 | 1.9 | 41.1 | 1.86 | Dawson and Fisher, 1969 |
| Verdin | *Auriparus flaviceps* | Desert | 7.0 | 0.8 | 37.8 | 5.33 | Wolf and Walsberg, 1996 |
| Common Nighthawk | *Chordeiles minor* | Tropical | 72.0 | 1.9 | 35.8 | 1.50 | Lasiewski and Dawson, 1964 |

**Table S7:** A summary of the three best performing conventional and phylogenetic generalised least squares (PGLS) models used to compare two measures of evaporative water loss (EWL); namely the inflection point in the relationship between air temperature (Tair) and EWL (Tewl, °C) and the slope of the line above this point (log10 EWL slope), in 34 species from across the globe, including data from Fynbos species at Blue Hill Nature Reserve (Table S6). All 34 species were compared in terms of the broad climatic zone in which the majority of their distribution is centred (Climate zone, (Kottek *et al.*, 2006). In each case, AICc (Akaike’s Information Criterion with correction for small sample sizes) values were compared to select the best-fitting models. In cases where AICc values were very similar (∆AICc< 2), the simplest model with the least explanatory factors was selected as the best model, following (Arnold, 2010). Shown in this table are each conventional and PGLS models for each physiological parameter with their respective AICc and ∆AICc (with respect to the best-fitting model) values. The model selected as the best model is presented in bold.

| **Analysis** | **Physiological parameter** | **Model** | **AICc** | **∆ AICc** |
| --- | --- | --- | --- | --- |
| Conventional | Tewl | **1) Tewl ~ log10 Mb+ Climate zone + log10 Mb x Climate zone** | 138.8 | 0.00 |
|  |  | 2)Tewl ~ Climate zone | 150.1 | 11.4 |
|  |  | 3) Tewl ~ log10 Mb+ Climate zone | 152.1 | 13.3 |
|  | log10EWL Slope | **1) log10 EWL Slope ~ log10 Mb + Climate zone + log10 Mb x Climate zone** | -37.4 | 0.00 |
|  |  | 2) log10EWL Slope ~ log10 Mb + Climate zone | -29.2 | 8.18 |
|  |  | 3) log10EWL Slope ~ log10 Mb | -26.0 | 11.3 |
| PGLS | Tewl | 1) Tewl ~ log10 Mb+ Climate zone + log10 Mb x Climate zone | 144.4 | 0.00 |
|  |  | 2) Tewl ~ Climate zone | 152.0 | 7.57 |
|  |  | 3) Tewl ~ log10 Mb+ Climate zone | 153.0 | 8.62 |
|  | log10 EWL Slope | **1) log10 EWL Slope ~ log10 Mb +Climate zone + log10 Mbx Climate** | -33.3 | 0.00 |
|  |  | 2) log10EWL Slope ~ log10 Mb+ Climate zone | -25.8 | 7.49 |
|  |  | 3) log10EWL Slope ~ log10 Mb | -24.9 | 8.40 |

**Table S8:** A summary of the outcomes of the best-fitting conventional and phylogenetic generalised least squares (PGLS) model for each of two measures of evaporative water loss (EWL); namely the inflection point in the relationship between air temperature and EWL (Tewl) and the slope of line above this point (log10 EWL slope), in 34 species for the Global Multispecies Comparative Analyses (Table S6). In all cases, the best perfoming model included the main effects of log10 Mb and Climate Zone, and the interaction effect of these two factors.

| **Analysis** | **Model** | **Coefficients** | **Estimate** | **s.e.** | **T** | **p** | **Significance** |
| --- | --- | --- | --- | --- | --- | --- | --- |
| Conventional | Tewl ~ log10 Mb + Climate zone + log10 Mb x Climate zone | Intercept (Desert birds) | 35.7 | 1.71 | 20.9 | 0.000 | *** |
|  |  | Slope (log10 Mb, Desert birds) | -2.51 | 1.10 | 2.28 | 0.030 | * |
|  |  | Intercept contrast (Temperate birds) | 7.53 | 2.82 | 2.67 | 0.012 | * |
|  |  | Intercept contrast (Tropical birds) | 4.02 | 2.55 | 1.58 | 0.125 |  |
|  |  | Slope contrast (log10 Mb x Temperate birds) | -9.14 | 2.09 | -4.37 | 0.000 | *** |
|  |  | Slope contrast (log10 Mb x Tropical birds) | -5.09 | 1.77 | -2.88 | 0.007 | ** |
|  | log10EWL Slope ~ log10 Mb+ Climate Zone + log10 Mb x Climate Zone | Intercept (Desert birds) | 1.12 | 0.14 | 8.14 | 0.000 | *** |
|  |  | Slope (log10 Mb, Desert birds) | -0.42 | 0.09 | -4.75 | 0.000 | *** |
|  |  | Intercept contrast (Temperate birds) | 0.64 | 0.23 | 2.82 | 0.009 | ** |
|  |  | Intercept contrast (Tropical birds) | -0.07 | 0.21 | -0.35 | 0.732 |  |
|  |  | Slope contrast (log10 Mb x Temperate birds) | -0.62 | 0.17 | -3.66 | 0.001 | ** |
|  |  | Slope contrast (log10 Mb x Tropical birds) | -0.02 | 0.14 | -0.17 | 0.870 |  |
| PGLS | Tewl ~ log10 Mb + Climate zone + log10 Mb x Climate zone | Intercept (Desert birds) | 36.4 | 2.59 | 14.03 | 0.000 | *** |
|  |  | Slope (log10 Mb, Desert birds) | 2.12 | 1.44 | 1.48 | 0.151 |  |
|  |  | Intercept contrast (Temperate birds) | 6.62 | 3.09 | 2.14 | 0.041 | * |
|  |  | Intercept contrast (Tropical birds) | 4.37 | 2.88 | 1.51 | 0.141 |  |
|  |  | Slope contrast (log10 Mb x Temperate birds) | -8.24 | 2.26 | -3.64 | 0.001 | ** |
|  |  | Slope contrast (log10 Mb x Tropical birds) | -4.97 | 1.82 | -2.73 | 0.011 | * |
|  | log10EWL Slope ~ log10 Mb+ Climate Zone + log10 Mb x Climate Zone | Intercept (Desert birds) | 0.86 | 0.21 | 4.17 | 0.000 | *** |
|  |  | Slope (log10 Mb, Desert birds) | -0.27 | 0.12 | -2.29 | 0.029 | * |
|  |  | Intercept contrast (Temperate birds) | 0.74 | 0.26 | 2.86 | 0.008 | ** |
|  |  | Intercept contrast (Tropical birds) | 0.14 | 0.23 | 0.61 | 0.546 |  |
|  |  | Slope contrast (log10 Mb x Temperate birds) | -0.71 | 0.19 | -3.72 | 0.001 | *** |
|  |  | Slope contrast (log10 Mb x Tropical birds) | -0.17 | 0.15 | -1.07 | 0.294 |  |

**References**

Ambrose SJ, Bradshaw SD, Withers PC, Murphy DP (1996) Water and energy balance of captive and free-ranging Spinifexbirds (Eremiornis carteri) North (Aves: Sylviidae) on Barrow Island, Western Australia. *Aust J Zool* 44: 107–117.

Arnold TW (2010) Uninformative parameters and model selection using Akaike’s Information Criterion. *J Wildl Manage* 74: 1175–1178.

Blomberg SP, Garland T, Ives AR (2003) Testing for phylogenetic signal in comparative data: behavioral traits are more labile. *Evolution (N Y)* 57: 717–745.

Burton CT, Weathers WW (2003) Energetics and thermoregulation of the Gouldian Finch (Erythrura gouldiae). *Emu* 103: 1–10.

Cade T, Tobin C, Gold A (1965) Water economy and metabolism of two estrildine finches. *Physiol Zool* 38: 9–33.

Dawson W (1954) Temperature regulation and water requirements of the Brown and Abert Towhees Pipilo fuscus and Pipilo aberti. *Univ Calif Publ Zool* 59: 81–123.

Dawson WR, Fisher CD (1969) Responses to temperature by the spotted nightjar (Eurostopodus guttatus). *Condor* 71: 49–53.

Garland T, Adolph SC (1994) Why not to do two-species comparative studies: limitations on inferring adaptation. *Physiol Zool* 67: 797–828.

Hackett SJ, Kimball RT, Reddy S, Bowie RCK, Braun EL, Braun MJ, Chojnowski JL, Cox WA, Han K-L, Harshman J, *et al.* (2008) A phylogenomic study of birds reveals their evolutionary history. *Science (80- )* 320: 1763–1768.

Jetz W, Thomas GH, Joy JB, Hartmann K, Mooers a O (2012) The global diversity of birds in space and time. *Nature* 491: 444–448.

Kottek M, Grieser J, Beck C, Rudolf B, Rubel F (2006) World map of the Köppen-Geiger climate classification updated. *Meteorol Zeitschrift* 15: 259–263.

Lasiewski RC, Dawson WR (1964) Physiological Responses to Temperature in the Common Nighthawk. *Condor* 66: 477–490.

Lighton JR (2008) Measuring Metabolic Rates: A Manual for Scientists. Oxford University Press, Oxford.

Maddison WP, Maddison DR (2011) Mesquite: a modular system for evolutionary analysis.

Matsuura K, Willmott C (2012) Terrestrial Precipitation: 1900-2010 Gridded Monthly Time Series (1900-2010)(v 3.01 Added 6/14/12). University of Delaware. University of Delaware.

Mbatha K, Downs CT, Penning M (2002) Nectar passage and gut morphology in the Malachite Sunbird and the Black-capped Lory: implications for feeding in nectarivores. *Ostrich* 73: 138–142.

McKechnie AE, Wolf BO (2010) Climate change increases the likelihood of catastrophic avian mortality events during extreme heat waves. *Biol Lett* 6: 253–256.

Orme D (2013) The caper package: comparative analysis of phylogenetics and evolution in R. *R Packag version 05 2* 1–36.

Paradis E, Claude J, Strimmer K (2004) APE: analyses of phylogenetics and evolution in R language. *Bioinformatics* 20: 289–290.

Schleucher E (1999) Energetics and body temperature regulation in two convergent dove species from extreme habitats. *Ornis Fennnica* 76: 199–210.

Smit B, Harding CT, Hockey PAR, McKechnie AE (2013) Adaptive thermoregulation during summer in two populations of an arid-zone passerine. *Ecology* 94: 1142–1154.

Smit B, McKechnie AE (2010a) Do owls use torpor? Winter thermoregulation in free-ranging pearl-spotted owlets and African scops-owls. *Physiol Biochem Zool* 83: 149–156.

Smit B, McKechnie AE (2010b) Avian seasonal metabolic variation in a subtropical desert: basal metabolic rates are lower in winter than in summer. *Funct Ecol* 24: 330–339.

Tieleman BI, Williams JB (1999) The role of hyperthermia in the water economy of desert birds. *Physiol Biochem Zool* 72: 87–100.

Tieleman BI, Williams JB (2002) Effects of food supplementation on behavioural decisions of hoopoe-larks in the Arabian Desert: balancing water, energy and thermoregulation. *Anim Behav* 63: 519–529.

Van den Berg EC, Plarre C, van den Berg HM, Thompson M (2008) The South African National Land Cover 2000, Report GW/A/2008/86. Agricultural Research Council-Institute for Soil, Climate and Water, Pretoria.

Walsberg GE, Wolf BO (1995) Variation in the respirometry quotient of birds and implications for indirect calorimetry using measurements of carbon dioxide production. *J Exp Biol* 198: 213–219.

Weathers W (1977) Temperature regulation in the Dusky Munia, Lonchura fuscans (Cassin) (Estrildidae). *Aust J Zool* 25: 193–199.

Weathers W (1997) Energetics and thermoregulation by small passerines of the humid, lowland tropics. *Auk* 114: 341–353.

Weathers W, Schoenbaechler D (1976) Regulation of body temperature in the budgerygah, Melopsittacus undulatus. *Aust J Zool* 24: 39–47.

Weathers WW, Caccamise DF, Url S (1975) Temperature regulation and water requirements of the Monk Parakeet , Myiopsitta monachus. *Oecologia* 18: 329–342.

Weathers WW, Greene E (1998) Thermoregulatory responses of Bridled and Juniper Titmice to high temperature. *Condor* 100: 365–372.

Whitfield MC, Smit B, McKechnie AE, Wolf BO (2015) Avian thermoregulation in the heat: scaling of heat tolerance and evaporative cooling capacity in three southern African arid-zone passerines. *J Exp Biol* 218: 1705–14.

Williams JB (1999) Heat production and evaporative water loss of dune larks from the Namib desert. *Condor* 101: 432–438.

Withers P, Williams J (1990) Metabolic and respiratory physiology of an arid-adapted Australasian bird, the Spinifex pigeon. *Condor* 92: 961–969.

Wolf BO, Walsberg GE (1996) Respiratory and cutaneous evaporative water loss at high environmental temperatures in a small bird. *J Exp Biol* 199: 451–457.
